# Microbial Identification, High-Resolution Microscopy and Spectrometry of the Rhizosphere in Its Native Spatial Context

**DOI:** 10.3389/fpls.2021.668929

**Published:** 2021-07-07

**Authors:** Chaturanga D. Bandara, Matthias Schmidt, Yalda Davoudpour, Hryhoriy Stryhanyuk, Hans H. Richnow, Niculina Musat

**Affiliations:** ProVIS-Centre for Chemical Microscopy, Department of Isotope Biogeochemistry, Helmholtz Center for Environmental Research (UFZ), Leipzig, Germany

**Keywords:** CARD-FISH, correlative chemical microscopy, Helium Ion Microscopy, London Resin White embedding, Rhizosphere, Secondary Ion Mass Spectrometry, Soil bacteria, Water-jet cutting

## Abstract

During the past decades, several stand-alone and combinatorial methods have been developed to investigate the chemistry (i.e., mapping of elemental, isotopic, and molecular composition) and the role of microbes in soil and rhizosphere. However, none of these approaches are currently applicable to characterize soil-root-microbe interactions simultaneously in their spatial arrangement. Here we present a novel approach that allows for simultaneous microbial identification and chemical analysis of the rhizosphere at micro− to nano-meter spatial resolution. Our approach includes (i) a resin embedding and sectioning method suitable for simultaneous correlative characterization of *Zea mays* rhizosphere, (ii) an analytical work flow that allows up to six instruments/techniques to be used correlatively, and (iii) data and image correlation. Hydrophilic, immunohistochemistry compatible, low viscosity LR white resin was used to embed the rhizosphere sample. We employed waterjet cutting and avoided polishing the surface to prevent smearing of the sample surface at nanoscale. The quality of embedding was analyzed by Helium Ion Microscopy (HIM). Bacteria in the embedded soil were identified by Catalyzed Reporter Deposition-Fluorescence *in situ* Hybridization (CARD-FISH) to avoid interferences from high levels of autofluorescence emitted by soil particles and organic matter. Chemical mapping of the rhizosphere was done by Scanning Electron Microscopy (SEM) with Energy-dispersive X-ray analysis (SEM-EDX), Time-of-Flight Secondary Ion Mass Spectrometry (ToF-SIMS), nano-focused Secondary Ion mass Spectrometry (nanoSIMS), and confocal Raman spectroscopy (μ-Raman). High-resolution correlative characterization by six different techniques followed by image registration shows that this method can meet the demanding requirements of multiple characterization techniques to identify spatial organization of bacteria and chemically map the rhizosphere. Finally, we presented individual and correlative workflows for imaging and image registration to analyze data. We hope this method will be a platform to combine various 2D analytics for an improved understanding of the rhizosphere processes and their ecological significance.

## Introduction

Soils are heterogeneous mixtures of various organic materials, mineral particles, and pores, which provide a matrix for plant growth and resource recycling by microbiome. Soil structure defines microenvironmental conditions and processes such as water retention, root penetration, microbial distribution, nutrient and gas exchange (Rabot et al., 2017; Kenney et al., 2019; Wilpiszeski et al., 2019). Though these individual aspects are extensively studied, correlative studies relating to multiple physical-chemical and biological interactions in the rhizosphere are limited. Particularly, the structural and biochemical complexity and spatial arrangement of microbes of the rhizosphere as the main driver of terrestrial biogeochemical cycles is little understood. To explore such relationships between multiple components (Ganther et al., 2020), it is crucial to characterize all soil components whilst keeping their structural and chemical integrity i.e., chemical composition and spatial organization of minerals, non-living organic matter and microbial counterparts such as bacteria, fungi, and plant roots during the analysis (Nunan et al., 2007; Mueller et al., 2013, 2019; Steffens et al., 2017; Vidal et al., 2018; Juyal et al., 2019, 2020; Fadel et al., 2020). The main reason that confines studies to a single discipline, rather than expanding to combinatorial characterization is associated with sample preparation. Multi-disciplinary analysis demands multiple compatibility requirements to accommodate for multiple techniques allowing the concomitant analysis of: (1) root (2) soil and (3) microbes in their spatial context. Nevertheless, a comprehensive chemical characterization coupled to microbial identification and distribution at spatial scales relevant to biogeochemical processes is necessary to achieve a systematic understanding of the key factors governing the self-organization of the rhizosphere (Nunan et al., 2007; Baveye et al., 2018; Juyal et al., 2019).

The correlation of multiple imaging techniques has received significant attention for soil research in recent years (Knierim et al., 2012; Saatz et al., 2016; Juyal et al., 2018, 2019; Gao et al., 2019; Schlüter et al., 2019; Ganther et al., 2020; Vetterlein et al., 2020). Using various techniques to characterize a single region of interest (RoI) (e.g., hot spot of microbial activity) can overcome the limitations of each individual technique. Thus combining different analytical capabilities will lead to in depth characterization of the sample and understanding of multiple processes within the rhizosphere. A combination of 2D chemical spectroscopy and microscopy provide new insights into the chemistry and organization of the rhizosphere at biologically relevant scales to uncover and quantify biogeochemical processes with spatial resolutions at micro- to nanometer scale (Juyal et al., 2019). For high-resolution chemical microscopy soil samples needs to be dehydrated and embedded in various matrixes as a preprocessing step to preserve and stabilize its structure under high vacuum analysis (Tippkötter and Ritz, 1996; Nunan et al., 2001; Bronick and Lal, 2005; Herrmann et al., 2007a,b; Juyal et al., 2019). Depending on the characterization technique, such a matrix can be gelatin (Olcott, 1960), sulfur (Lehmann et al., 2005; Kinyangi et al., 2006), water glass (Gobinath et al., 2019; Szoboszlay and Tebbe, 2020) or a polyester, epoxy, or acrylic resins (Tippkötter and Ritz, 1996; Schlüter et al., 2019). Among these, Araldite 502 is a widely used epoxy resin for soil embedding because it is fast out-gassing, vacuum compatible and can easily be polished to obtain a smooth surface for surface analysis (Tippkötter and Ritz, 1996; Herrmann et al., 2007a). Upon availability of a compatible sample, Helium Ion Microscopy (HIM), Scanning Electron Microscopy (SEM) with Energy-dispersive X-ray analysis (SEM-EDX), Time-of-Flight Secondary Ion Mass Spectrometry (ToF-SIMS), nano-focused Secondary Ion mass Spectrometry (nanoSIMS), confocal Raman spectroscopy (μ-Raman), and fluorescence microscopy, are promising for comprehensive characterization of specific chemical and microbial interactions within the rhizosphere. The capabilities of these instruments related to soil research are briefed in the SI (Supporting Information) section.

Of particular interest in rhizosphere research, is the inherent microorganisms, key players of the carbon and nutrient recycling processes (Herrmann et al., 2007b; Pett-Ridge and Firestone, 2017). For their correct identification and quantification, fluorescent dyes should stain all microbial cells independent of their physiological state and metabolic activity (Zweifel and Hagstrom, 1995; Klauth et al., 2004). In a complex environmental sample, non-specific binding sites for fluorescence stains are significantly higher to the number of specific DNA binding sites available (Nunan et al., 2001). Moreover, fluorescent signals from non-specific site bound fluorescent dyes are often similar in shape, size and intensity to those of bacterial DNA bound fluorescent dyes. This similarity of fluorescent signals between DNA-bound dyes and non-specific site bound dyes is leading to false positive identification of microbes (Klauth et al., 2004). Therefore, bacterial counts of environmental samples will not be accurate with non-specific staining of DNA (Nunan et al., 2001; Klauth et al., 2004; Eickhorst and Tippkötter, 2008). Thus, for an accurate identification and counting, bacteria are usually detached (Klauth et al., 2004) for molecular biological and physio-chemical procedures to identify different organisms in their abiotic environments. As soil environments are often disrupted for the extraction and quantification of microbial cells, nucleic acids or proteins (Ganther et al., 2020; Holz et al., 2020; Szoboszlay and Tebbe, 2020), such approaches leads to loss of essential biological information, such as morphology of cells, association with other organisms e.g., aggregates, biofilms and their spatial organization (Kenney et al., 2019). Therefore, these techniques cannot be used in a correlative microscopic approach. CARD-FISH method can overcome issues associated with non-specific binding and improve the visualization of bacterial cells in a resin embedded sample (Gros and Maurin, 2008; Schimak et al., 2012). However, widely used resins for soil embedding, for instance, Araldite, Epon, and Lowicryl to name a few, are not compatible for hybridization steps in CARD-FISH. Their hydrophobicity once hardened, which prevents the probe penetration step, thereby does not allow bacterial identification (Knierim et al., 2012). Therefore, simultaneous characterization of microbes and correlation with the physio-chemical data within the intact rhizosphere soil is limiting (Gros and Maurin, 2008). Thereby an embedding method allowing successful microbial identification *in situ* and simultaneous high-resolution physio-chemical characterization is urgently needed for comprehensive understanding of microbe-soil-root functions.

The focus of this work was to develop an embedding method which appropriately identify bacteria and, at the same time, allow chemical composition analysis of an undisturbed rhizosphere using correlative microscopy and spectrometry approach. However, preparation of a soil sample for various 2D and 3D characterization techniques with minimal destruction to the rhizosphere is a challenging task due to the sample size and the heterogeneous composition of organic and inorganic components. High spatial resolution techniques require mechanically stable and absolutely dry samples that are compatible with the required (ultra) high vacuum conditions. In this study, we developed a sample preparation method and a correlative imaging workflow for soil sub-samples to comprehensively characterize the spatial organization of roots, minerals and bacteria within the rhizosphere of *Zea mays*. Our approach consists of fixation and dehydration of the soil sample followed by impregnation with the vacuum- and CARD-FISH- compatible London Resin White (LR white), sectioning with a waterjet to avoid polishing of the surface, and finally correlative imaging with multiple microscopic and spectroscopic techniques. Microbial identification keeping native spatial arrangement within the rhizosphere by CARD-FISH technique in a correlative high-resolution chemical microscopy, and mass spectrometry workflow is a unique feature of our approach.

## Materials and Methods

### Sample Preparation

#### Soil Column Preparation and Subsampling

**FIGURE 1:**
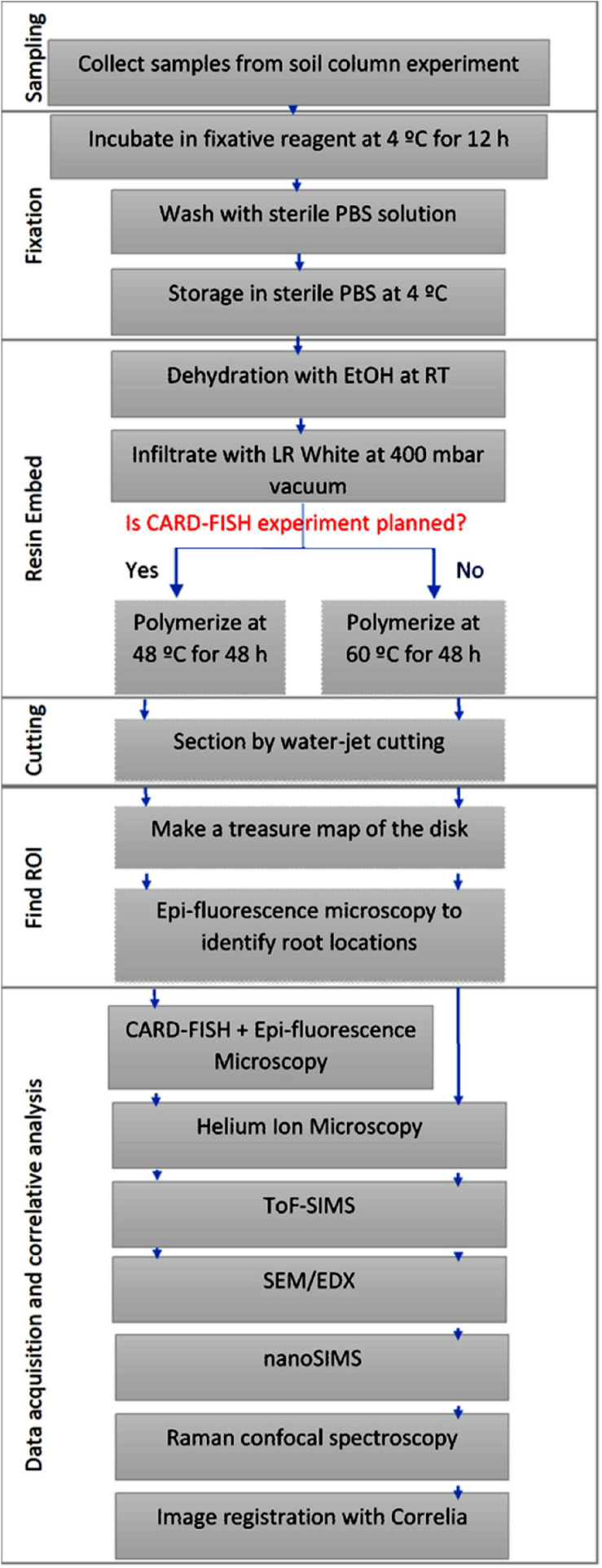
Schematic representation of the sample preparation, image registration and correlative analysis.

The soil substrate used is loam textured Haplic Phaeozem soil collected at Schladebach, Germany. Final soil column substrate corresponds to a bulk density of 1.26 g cm^–3^ (pH 6.21, sand 33.2%, silt 47.7%, clay 19.1%). Concise methodology on sampling and preparation of soil columns (Ganther et al., 2020), substrate properties, and detailed subsampling method is documented elsewhere (Vetterlein et al., 2020). Soil layers are carved into small metal cylinders (16 mm diameter) and to prevent falling off of the soil top and bottom of these sub-samples were covered with a mesh and tightened with cable ties. In doing so, five sub-samples per depth were sampled out of which one cylinder was selected for resin embedding to prepare for 2D chemical and microbial imaging techniques. Acquired sub-samples were then fixed with either 2% paraformaldehyde or Karnovsky fixative (2% Paraformaldehyde, 2.5% Glutaraldehyde in 0.1M Buffer), for overnight at 4°C (Garland et al., 1979; Mount et al., 1997). Samples were rinsed twice with 1% PBS solution and stored at 4°C in a 1% PBS solution until resin embedding. Figure 1 represents the sample preparation work flow. Appearance of sample at different steps of the workflow is presented in the Supporting Figure 1.

#### Resin Embedding

Fixed samples were initially washed twice with 25 mL mili-Q water by immersing in for 30 min each. The washed samples were placed in a 50 mL Eppendorf tube filled to 5 mL level with 2.85–3.45 mm size glass beads (Carl Roth GmbH, Germany) and dehydrated by immersing in increasing ethanol (Sigma-Aldrich) concentrations of 30, 50, 70, 80, 90, 95, and 99% for one hour each. Further on, samples were subjected to 100% ethanol (water-free) in three consecutive steps: (1) 1 h; (2) transferred to a new 50 mL Eppendorf tube (filled with fresh 2.85–3.45 mm size glass beads up to 10 mL level) and slowly pumped till 400 mbar vacuum and left for 14–16 h (overnight); (3) and immersed in a fresh 100% ethanol for 1 h before resin infiltration step.

London Resin (medium) (LR white) (Agar Scientific, United Kingdom) infiltration was carried out with consecutive steps of increasing EtOH:Resin mixture (vol/vol) 3:1, 3:2, 1:1, 2:3, 1:3, resin 1 h each step. Next, the samples were treated with 100% resin three times for 1 h each. The sample was then transferred to a fresh 50 mL Eppendorf tube, filled with glass beads (to 5 mL level) and then filled up to 30 mL level with LR white resin, and kept for 14–16 h (overnight) under vacuum at 400 mbar, with continuous purging with Ar gas. Care was taken to slowly reduce the vacuum to 400 mbar. Finally, tubes were exposed to Ar gas for 2 min, tightly sealed and samples cured in a water bath for 48 h at either 48°C or 60°C.

The sample was dehydrated with ethanol and then substituted gradually with resin by increasing resin concentrations. Gradual dehydration and infiltration reduce the potential of causing any distortion to ultrafine cellular structures and the microbial colonies and less lipid component are removed (Kenney et al., 2019). We have used ethanol instead of acetone as this is weaker solvent compared to acetone and therefore less prone to extract cell components such as lipids. Glass beads were used during dehydration and embedding steps to lift the sample from the surface and keep it straight during the process. This stops air trapping at bottom and helps exchanges from the top and bottom surfaces to increase the embedding efficiency. This also helps positioning sample during the water-jet cutting. Care was taken at every step to make sure the transfer of solvents take place slowly and to prevent air trapping in between these glass beads, which can result in poor infiltration.

#### Water-Jet Cutting

The cured resin embedded samples were securely mounted in a horizontal plane on to the waterjet cutting system (Premium Cut 3D; StM Waterjet GmbH, Salzburg, Austria) using an ad-hock designed clamp. A combination of pressurized water and abrasive garnet particles cut the sample at predefined positions. Initially, abrasive particles of two mesh sizes (Mesh-50 of 300 μm in diameter and Mesh-200 of 75 μm diameter) and different settings are used to optimize the final surface roughness using pure resin. These settings and resulted roughness are listed in the SI section. The embedded soil sample was cut with the settings that resulted in minimum surface roughness. Care was taken not to cut the soil sub-sample completely to prevent it falling into water bath during the cutting procedure. Once multiple cuttings were performed and dismounted from the instrument, samples were washed three times by immersing in Mili-Q^®^ water for 10 minutes each. Individual disks (1 mm thick) were cracked to separate and air-dried before stored in a gel-box (Gel-Pak^®^) until further analysis.

#### CARD-FISH

The LR white embedded soil disks were divided into multiple pieces for hybridization using a mixture of the following probes: EUB 338I, II, and III (Amann et al., 1990; Daims et al., 1999), to target a wide range of soil bacteria in the sample. This protocol follows Pernthaler et al. (2002) with following modifications. Embedded soil disks were permeabilized in a Lysozyme solution (10 mg/mL in 0.05 M EDTA, pH 8.0; 0.1 M Tris-HCl, pH 7.5) for 1 h at 37°C, followed by washing with ultrapure (mili-Q) water and further treatment with 60 U mL^–1^ Achromopeptidase for 30 min at 37°C. The enzymatic permeabilization was followed by 3× washing with mili-Q water and treated for inactivation of endogenous peroxidases in 0.15% H_2_O_2_ in methanol (vol/vol) for 30 min., and further on followed by washing 3× with ultrapure water at room temperature (RT) and dehydrated by increasing EtOH series 50, 70, and 96%, 30 s each and air dried. The hybridization was done overnight (app. 20 h) at 35°C in a hybridization buffer containing (0.9 M NaCl, 40 mM Tris-HCl, 10% (w/v) dextran sulfate, 0.01% (w/v) sodium dodecyl sulfate (SDS), 10% blocking reagent, 1× Denhard’s reagent, 0.26 mg mL^–1^ sheared salmon sperm DNA, 0.2 mg mL^–1^ yeast RNA, 5 M NaCl 3.6 mL, and 50% formamide. The HRP-labeled probes (Biomers, Ulm, Germany) applied are specific for bacteria were used at a concentration of 0.166 ng mL^–1^ (HRP-probe stock solution of 50 ng mL^–1^ diluted 1:300 v/v in hybridization buffer). Following hybridization, resin embedded soil pieces were incubated in 50 mL of pre-warmed washing buffer containing 19 mM NaCl, 5mM EDTA (pH = 8.0), 20mM Tris-HCl (pH = 7.5), and 0.01% SDS for 15 min at 37°C. After washing, samples were incubated for 15 min at RT in 1 × PBS (pH = 7.6) to equilibrate the HRP-labeled probe. Subsequently, tyramide deposition was performed by incubation for 30 min at 46°C in the dark in amplification buffer containing 1 × PBS, 2M NaCl, 0.1% (w/v) blocking reagent, 10% (w/v) dextran sulfate, 0.0015% (v/v) H_2_O_2_, and 1 μg mL^–1^ Alexa 594-labeled tyramides (ThermoFisherScientific, Waltham, MA, United States). Afterwards, samples were rinsed in 1 × PBS for 10 min at RT followed by counterstaining with 4’,6-diamidino-2-phenylindole (DAPI) 1μg mL^–1^ for 10 min at RT, washing in ultrapure water and air dried. Samples stored at −20°C until imaging.

### Correlative Imaging

#### Light Microscopy

The embedded and cross-sectioned soil disk was mapped entirely with reflected light microscopy, using a WITec alpha 300 confocal Raman microscope (WITec GmbH, Ulm, Germany) equipped with the true surface^®^ option to make an overview montage. The acquired 2704 individual mosaic images with 136 stack layers were stitched in auto mode with continuous movement algorithm by WITec project five (5.2) software to make a single figure of 15.857 mm × 15.857 mm. This overview is the treasure map to locate RoI for various subsequent micro-analyses. As roots could not be efficiently located by this method, epifluorescence imaging was adapted to find areas with roots for further analysis for other imaging techniques.

#### Epifluorescence Microscopy

In order to swiftly locate roots in the soil advantage of their autofluorescence was taken by mapping the resulted soil-disks with epifluorescence microscopy (Zeiss AxioImager.Z2). The microscope was equipped with an HXP R 120W/45C UV Hg-vapor lamp, Colibri.2 LED illuminations (590 nm, 470 nm and 365 nm) and imaged with objective lens 20X (EC Plan-Neofluar/0.50 M27). Fluorescence DAPI filter set was used to visualize autofluorescence of the root. Images were acquired with a color CCD camera connected to an imaging software (Zeiss AxioVision).

Catalyzed Reporter Deposition-Fluorescence *in situ* Hybridization (CARD-FISH) hybridized samples were imaged with fluorescent filter sets [DAPI (365 nm), DsRed (590 nm)], using 20X (NA = 0.50) and 50X air objectives (NA = 0.55) and a black and white CCD camera (AC MR R3) connected to Zeiss AxioVision. Acquired images were false colored to respective channel, DAPI in blue and DsRed in orange.

#### Helium Ion Microscopy (HIM)

After epifluorescence microscopy mapping RoIs were imaged with HIM (Zeiss Orion NanoFab, United States). The reason for using HIM instead of SEM is that this surface-sensitive method can be considered almost non-destructive: Neither a significant damage by the ion beam has to be expected (only about 30 He^+^ -ions are implanted per pixel) nor does it require a metal- or carbon-coating of the surface, as is needed for SEM to avoid charging. HIM imaging was carried out with He^+^ at 25 kV acceleration voltage and a beam current of 0.1 to 0.3 pA. Secondary electron images were collected in a quadratic field-of-view of 300 μm. The dwell time amounted to 0.5 μs and 32 times line averaging was done to reduce noise. For charge compensation an electron flood gun was used during imaging. In total nine fields-of-view were acquired with 25% overlap and stitched together using either the pairwise stitching (Preibisch et al., 2009) or mosaicJ (Thevenaz and Unser, 2007) plugins in Fiji to create a larger map of the RoI. The obtained HIM microscopies of the RoI are rich in features and thus serve as an ideal base for the registration of other modalities.

#### ToF-SIMS Experiment

RoIs of soil disks were analyzed with a TOF-SIMS 5 instrument (IONTOF GmbH, Münster) employing 30 keV Bi_3_^+^ cluster ions (LMIG NanoProbe source) as primary projectiles. Secondary ions were analyzed in either negative or positive extraction mode depending on the experiment. With a 100 μs period of primary pulse repetition, the sample surface was exposed to 0.05 pA of Bi_3_^+^ probe and the secondary ion species were detected within a 12-1200 Da range in Delayed-Extraction mode allowing for 120–250 nm lateral resolution and about 5000 MRP (Benettoni et al., 2019). Analyses of 250 × 250 μm^2^ size RoI’s were performed by rastering the Bi_3_^+^ probe randomly across 2048 × 2048 px raster-pattern reaching 2 shots per pixel in 28 min of single analysis frame. After acquisition of each analysis frame, an area of 500 μm × 500 μm or 800 μm × 800 μm around the analysis crater was sputtered with either (i) 75 nA of 1 keV Cs^+^ or (ii) 250 nA of 1 keV O_2_^+^ beam in 200-400 frames for 3–7 min to analyze the complex heterogeneous sample in depth. Sputtering with Cs^+^ ion beam was examined in negative as well as positive extraction mode, whereas sputtering with O_2_^+^ was examined only in the positive extraction mode. To compensate for surface charging of insulating uncoated samples, flooding with Ar gas (partial pressure 2 × 10^–6^ mbar in analysis chamber) was employed in combination with 21 eV electrons from pulsed neutralizing electron gun (NEG) during the analysis. Between 80 and 200 planes were acquired per analysis. The lateral drift, mass-shift corrections and further data evaluation steps were performed with the Surface Lab software (Version 7.1, ION-TOF GmbH, Germany) to generate peak lists and corresponding ion distribution maps. Negative mass spectra were calibrated using the mass peaks assigned to H^–^, C^–^, CH^–^, O^–^, and OH^–^. The peak lists were generated by the automatic peak search program between 0–1000 m/z mass range. This step resulted in 687 mass peaks for multivariate analysis. Initially, PCA was done to determine the number of principal components for the analysis. Though 155 PCs were suggested for this sample, we have only selected 10 PCs for further analysis in MCA to determine their corresponding spectra.

#### NanoSIMS Experiment

The resin-embedded rhizosphere samples were mounted on a sample holder, 5 connecting bridges were made for better conductivity (see Supporting Information) and coated with 20 nm Au/Pd (80/20%) layer to provide a conductive surface and analyzed with a nanoSIMS 50L instrument (CAMECA, AMETEK) in either negative or positive extraction modes.

In the negative extraction mode, 16 keV Cs^+^ ions with a beam current of 3 pA beam were employed to analyze 95 × 95 μm^2^ areas in sawtooth raster of 512 × 512 pixels. The dwell time was 2 ms/pixel and a field aperture of 300 μm diameter was used. Prior to the analysis, the targeted field of view (FoV) of 150 μm × 150 μm was pre-implanted with 200 pA Cs^+^ beam for 20 min. Mass Resolving Power (MRP = M/dM) above 7000 was achieved with 20 μm × 140 μm (width × height) entrance slit, 200 μm × 200 μm aperture, 40 × 1800 μm exit slits and the energy slit cutting-off 20% of ions at their high-energy distribution site, for the collected secondary ion species (^12^C_2_^–^, ^13^C^12^C^–^, ^12^C^14^N^–^, ^13^C^14^N^–^, ^31^P^–^, ^32^S^–^, and ^31^P^16^O_2_^–^).

In the positive extraction mode, a 5 nA O^–^ beam was pre-implanted in an area of 150 μm × 150 μm for 30 min. The analysis was done with 20 pA of 16 keV O^–^ ion beam in 100 × 100 μm^2^ sample areas (within those pre-implanted) in 512 × 512 pixels with dwelling time of 2 ms/pixel and the field aperture of 300 μm diameter. Mass Resolving Power (MRP = M/dM) above 6000 was achieved with a 20 μm × 140 μm (width × height) entrance slit, 350 × 250 μm aperture, 40 × 1800 μm exit slits and the energy slit cutting-off 10% of ions at their high-energy distribution site, for the collected secondary ion species (^11^B^+^, ^24^Mg^+^, ^27^Al^+^, ^31^P^+^, ^40^Ca^+^, ^55^Mn^+^, and ^64^Zn^+^).

During the analysis 30-100 plains were acquired, and corrected for lateral drift and accumulated using the Look@NanoSIMS software (LANS) (Polerecky et al., 2012). Individual data files were further processed by the OPENMIMS ImageJ plugin to extract only 5 image planes and to create a combined field of view showing larger area of rhizosphere using mosaic J plugin (Thevenaz and Unser, 2007) in Fiji.

#### SEM-BSE and SEM-EDX

In order to obtain mineralogical information of a RoI selected based on the fluorescence micrograph, the surface was analyzed with a field-emission SEM (Zeiss Merlin VP Compact) in BSE-mode and coupled to an EDX spectrometer (Bruker Quantax XFlash 5060F), respectively, at an electron acceleration voltage of 11.8 kV and a beam current of about 250 pA. Firstly, the surface was scanned using the in-lens electron detector negatively biased at 958 V to completely suppress secondary electrons but allow high-energy back-scattered electrons to be detected. This resulted in high-resolution images of the rhizosphere providing a material contrast sufficient to differentiate minerals, root cells and resin infiltrations. To obtain more specific information about the elemental composition SEM-EDX maps were collected using a Bruker Quantax XFlash 5060F energy-dispersive X-ray spectrometer (Bruker Nano GmbH Berlin, Germany). The resulting element-distribution maps were background-corrected by removing the bremsstrahlung using a physical model implemented in the software Bruker Esprit 1.9. The SEM-BSE as well as the SEM-EDX maps are conveniently registered onto the helium ion micrographs (for SEM-EDX using the Si channel for registration of the entire set) employing automatic rigid mutual-information method implemented in the ImageJ plug-in Correlia (Rohde et al., 2020).

#### Confocal Raman Spectroscopy

Selected root-soil interfaces were analyzed with the same WITec alpha 300 confocal Raman microscope (WITec GmbH, Ulm, Germany) used to acquire the overview light micrograph serving as treasure map. For excitation a solid-state laser emitting in the infrared (785 nm) was used. The analysis was carried out at a laser power of 0.5 mW and spectra were acquired with a grating monochromator (600 g/mm) and CCD camera detection. Mapping was done in an area of 160 μm × 160 μm, with 0.25 μm pixel-size. The objective lens was a Zeiss LD plan-NEOFLUAR 20X air objective lens with 0.4 numerical aperture, allowing resolution of less than 1 μm. The WITec project five (5.2) software was utilized for the data processing. After cosmic ray removal and background subtraction, the automated statistical evaluation of all spectra by *k*-means cluster analysis resulted in spectral unmixed images which provided an insight into the chemical composition of the sample.

Soil minerals were analyzed with the same system but using 532 nm wavelength and 33 mW laser power instead. An area of 170 μm × 170 μm was scanned with an integration time of 10 s and 85 point per x,y direction of scan resulting 861 s per line scan using the 20X air objective lens.

## Results and Discussion

Soil samples for high-resolution microscopy must be dry, stable, compatible with ultra-high vacuum (10^–8^–10^–10^ Torr) and conductive unless charge compensation is available. As such, soil samples containing water must be pretreated prior to characterization. On top of that smooth and highly polished surfaces are expected to obtain optimal conditions for their characterization. Therefore, the preparation of soil samples for high-resolution microscopy involves stabilizing biological components (fixation), removing water (dehydration), resin-embedding, and polishing (Herrmann et al., 2007b).

Fixation, dehydration and resin embedding are important initial steps to preserve the original morphology of living microbial cells and plant roots in their original condition (Pathan et al., 2008; Kenney et al., 2019). Therefore, we fixed the sub samples immediately after collecting from the soil columns to limit structural alterations that may occur to roots and microbes during sample preparation and storage. Considering the complexity and size of the rhizosphere samples, high pressure freezing and freeze substitutions, which appear to be advantageous over chemical fixation, are not compatible (Herrmann et al., 2007b). Therefore, we adapted the Karnovsky fixative (Garland et al., 1979; Mount et al., 1997) in our sample preparation. Aldehyde-based fixatives preserve cells by crosslinking proteins, which results in the stabilization of the cellular fine structure. It is crucial to select the right fixation method when chemical characterization of the sample is planned. Several studies show that the choice of fixative and their time of action can affect the overall chemistry of the sample (Pathan et al., 2008, 2010; Musat et al., 2016; Fiedler et al., 2018). Widely used fixatives such as glutaraldehyde and ethanol can cause significant changes to the Raman spectra of bacteria indicating chemical alterations, whereas formaldehyde and sodiumazide were better at preserving spectral features (Fiedler et al., 2018). As rhizosphere consists of soil, root, bacteria and other organic and inorganic material, various components can be studied, and it is not trivial to adapt a single fixative method. We have used ethanol instead of acetone, to minimize the membrane damages. Another critical factor to be considered is the choice of resin as this could change the chemistry of the sample (Yeni et al., 2006). The ‘*ideal*’ resin may only introduce minimal physiological and chemical artifacts to the test sample, and it has to be compatible for multiple imaging requirements of various analysis methods. Overall, it is important to note that the fixation and resin embedding steps have to be carefully selected depending on the specific analysis planned. The soft LR white resin we choose is used to embed and preserve microbial cells in various tissues, the environmental samples in correlation with FISH and TEM to study functions of microbes in natural systems (Knierim et al., 2012; Schimak et al., 2012; Susanne Kramer et al., 2020). However, LR white has not yet been used for soils or rhizosphere samples in high resolution workflows where bacteria-plant-organic-inorganic interphases are correlatively characterized.

**FIGURE 2:**
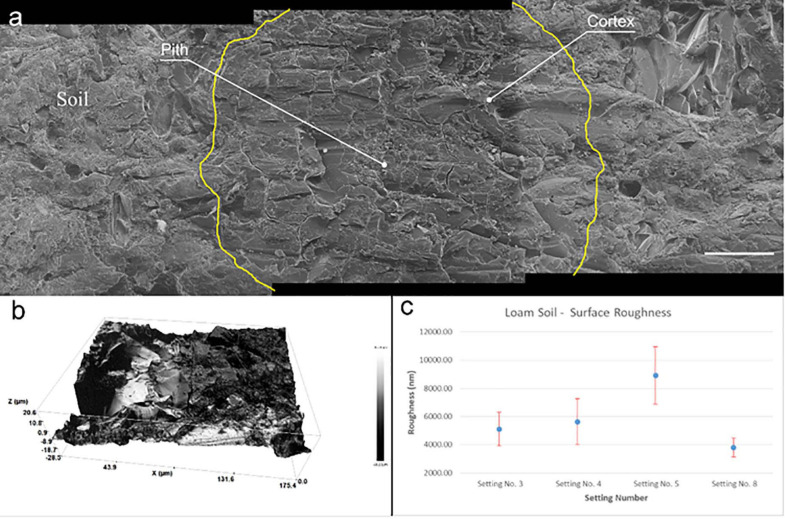
Surface roughness of embedded soil sample. **(a)** Three helium ion micrographs are stitched showing the quality of embedding and surface roughness of a root after water jet cutting. Root cell is located in the middle image and outlined in yellow for easy recognition. Root cells and soil-mineral interface are well infiltrated by the embedding procedure. Scale bar 50 μm **(b)** Surface profile of a root area measured by profilometer before microscopy. Rz = 49.8 μm **(c)** Resulted surface roughness of embedded soil once cut with different parameters by water-jet.

The helium ion micrographs in Figure 2c demonstrate the quality of embedding with LR white using this procedure. Overall, the quality of infiltration is satisfactory as neither holes representing poorly infiltrated areas nor cracks representing changes in the soil structure are noticed in the resulted samples after water-jet cutting. There were no gaps found at the root-soil interface, and minerals are in contact with the outer root wall and minerals of different sizes are held by the cured resin. The root cells in the center of the image are well infiltrated by resin without vicinity of pores, holes or non-cured areas. HIM images mainly show a topography contrast with pronounced edges. The weak contrast in Figure 2c is an indication for the low number of edges noticeable, indicating consistent infiltration of rhizosphere. Consistency in resin infiltration and curing are mainly due to the low viscosity of LR white compared to common polyester (380–800 mPas) and epoxy (200 mPas) resins. This allows LR white resin to effectively penetrate through the soil pore network and into root cells to preserve rhizosphere for microscopic analysis in (ultra-) high vacuum conditions (Tippkotter and Ritz, 1996). However, within the root, sub-micron scale space between cell wall and cell cytoplasm is evident. This is due to the shrinkage of resin during the curing process. This separated space is also apparent between the soil and the Al cylinder of the soil-sample (see Supporting Figure 12). However, LR white shows the minimum shrinkage of 2% during impregnation and curing compared to most commonly used epoxy and polyester resins, which showed 6.4% and 7.5% shrinkage, respectively (Tippkotter and Ritz, 1996). LR white is sensitive to oxygen, therefore, samples were processed in a desiccator purged with Ar during infiltration and sealed when curing. More often, traditional soil-related embedding demands hard-plastic resin to polish the surface, where soft LR white resin does not fit to prevent the smearing during cutting and polishing steps (Tippkotter and Ritz, 1996). Softness can lead to excessive smearing and delocalization compared to hard resins during cutting and polishing. Consequently, we have adapted water-jet cutting procedure to slice the embedded soil and avoided polishing.

### Water-Jet Cutting and Surface Roughness

As the RoI of the rhizosphere is at the microscale, variations in the surface roughness might affect the nanoSIMS and ToF-SIMS measurements. To achieve a suitable surface roughness facilitating characterization with multiple analytical instruments, we have optimized water-jet cutting parameters. Roughness was measured using an optical profilometer (see Supporting Table 1) and achieved the minimum roughness of 40.0 ± 5.00 nm (Figure 2b). Figure 2b shows the resulted 3D roughness profile of the area where we have carried out correlative microscopic analysis. However, we have noticed roughness of embedded soil is random and highly changes locally compared to pure resin. Our results demonstrates that an Rz value of approximately 50 nm (Figure 2a) can provide comprehensive chemical measurements of the rhizosphere. Moreover, water-jet approach can be used to gain multiple disks with 1 mm thickness (see Supporting Figure 1-6) that allow to gain multiple cross-sections from single sub-soil sample. Thereby if the need arises, multiple planes along Z direction can be characterized, which is an advantage for a comprehensive understanding of the chemical gradients within the rhizosphere.

Water-jet is a versatile cutting technique adopted in various industries to cut heat-sensitive samples without resulting rough, barring edges. As an additional advantage, water-jet cut avoids heat generation during the cutting. Thereby, avoid possible heat damage for bacterial ribosomes, which might affect the CARD-FISH experiment. Further, this process only uses water and sand and does not produce any toxic gases during processing. Thereby environmentally friendly and can be directly discharged.

Surface imperfections on the resulted surface scatters light and decrease the quality of optical images of large fields of view. Therefore, polishing is a regular step adapted to prepare soil samples to achieve quality of visual images with fields of views of mm to cm scales. Yet to achieve flat surfaces without scratches or furrows are difficult with samples like soil with a mixture of components of varying hardness. Polishing can also increase surface furrows and deterioration of soft crystals, remove attached bacterial cells on the minerals, smearing, and unknown manipulation of the sample at the micro to nano scale. Therefore, we avoided the polishing of the surface, thereby, surface smearing and dislocation of materials at microscales by adapting water-jet cutting during soil sample preparation.

### Selecting Region of Interest (Root/Bacteria) by Light Microscopy

**FIGURE 3:**
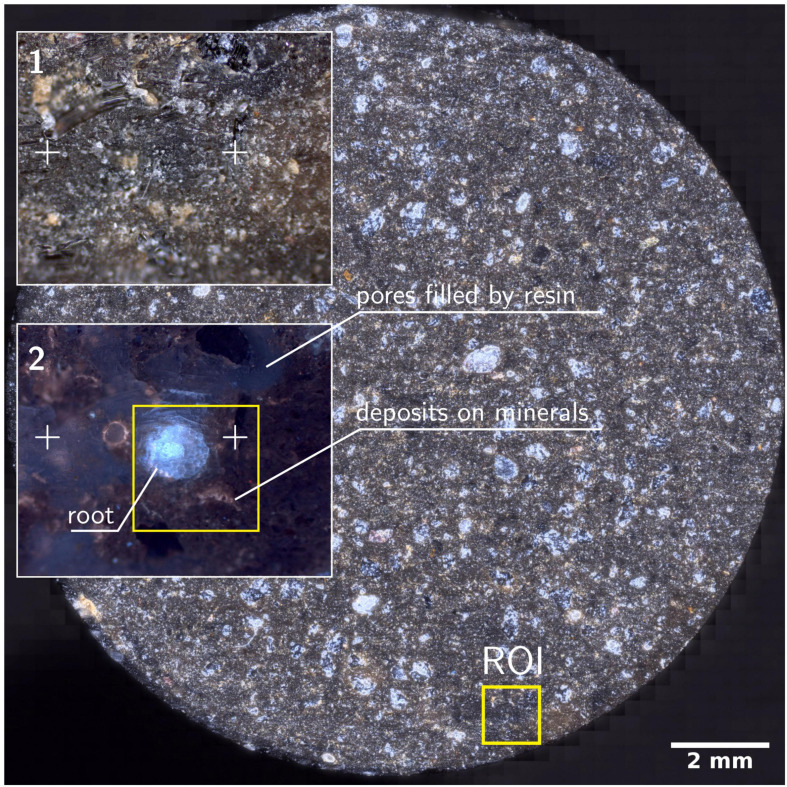
Resulted disk of soil column after water-jet cutting. Disk is a stitched image from 2704 individual images at darkfield microscopy mode. Insets 1-2 shows the magnified RoI marked in the soil disk. Crosses are surface features used to identify the RoI under different microscopes. (2) Epi-fluorescence micrograph of RoI showing the root, soil and minerals. Square marked in yellow is the RoI for high-resolution microscopy under HIM, ToF-SIMS, SEM-EDX, nanoSIMS and μ-Raman.

Bright- and darkfield reflective images resolved resin from mineral particles but cannot be used to identify roots or bacterial colonies in the rhizosphere (Figure 3). Homogeneous resin infiltration makes it challenging to select the RoI, which is a root in this study. Therefore, fluorescence microscopy is used to locate the RoI for further high-resolution microscopy. Epifluorescence microscopy under UV captures autofluorescence of embedded roots (cyan), and resin (dark blue), and minerals in dark color (Figure 3, inset), differentiating them from each other. The dark blue colored resin is the soil matrix’s air pores, now infiltrated by resin in this procedure. The selected area for further analysis is well focused, while a dominant scratch and bottom section of brightfield and fluorescence images are blurred. As the sample is not polished, and the depth of focus is out of the objective lens focus range, blurred sections appear. However, the root and the surrounding microscale area is in focus and is sufficient to obtain a clear image. Therefore, avoiding of polishing step has not influenced the image quality within RoI. There is a prominent tan colored border that is visible in some parts around the root and minerals. These correspond to Ca and P in EDX and nanoSIMS micrographs (Figure 3).

### Chemical Microscopy

#### ToF-SIMS

**FIGURE 4:**
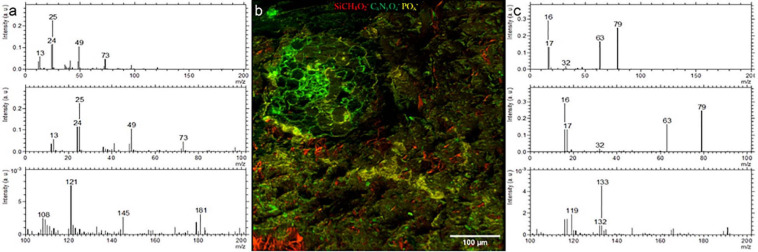
ToF-SIMS analysis of a Rhizosphere embedded sample. **(a)** Spectrum of LR white **(b)** Molecular fragment distribution related to SiCH_4_O_2_^−^, CNO^−^ and PO_*x*_^−^ **(c)** spectrum of PO_*x*_^−^ rich areas.

We could identify the resin, minerals and the root clearly using the negative ion extraction mode analyzed by PCA-MLS (Figure 4). Soil matrix was identified by the C_*x*_H_*y*_O_*z*_^–^ and SiCH_4_O_2_^–^ fragments and the cell walls of the root are identified by CN^–^ and CNO^–^fragments (Figure 4b). There are CNO^–^ fragments scattered randomly within soil matrix, which are indicated as green color tiny spots in the Figure 4. These may correspond to plant exudates or organic matter within the soil matrix. A pore area immediately above the root is filled with resin and appear separate to the root and soil matrix. LR white resin is composed of C, H, and O (see Supporting Figure 3 for EDX spectra) and therefore, resin related fragments can be distinguished by the C^–^(m/z = 12), CH^–^(m/z = 13), C_2_^–^(m/z = 24), C_2_H^–^(m/z = 25), CH_2_^–^ and O^–^ fragments (Figure 4a). Given the availability of other unknown hydrocarbon molecules within the soil matrix, it is difficult to distinguish resin C_*x*_H_*y*_O_*z*_^–^ fragments from the organic molecules which may be already present within soil matrix using currently available PCA methods. Nevertheless, with PCA we could easily distinguish root, PO_*x*_^–^ (Figure 4c) and minerals (see Supporting Figure 4). However, with future improvements in the complex analysis methods, and with an availability of a correlative sample preparation method, distinguishing biomolecules from LR white resin may be possible (Fadel et al., 2020).

On the root surface PO_*x*_^–^ deposition was recognized, on one side of the outer root wall, which spreads into the soil matrix in the analyzed plane, and correlates with nanoSIMS, EDX, and fluorescent image (UV) data. These precipitations may be related to the physio-chemical interactions between root’s nutrient uptake mechanisms (Abel, 2017). PO_*x*_^–^ precipitations were also noticed in soil matrix and are likely to occur as P anions highly associated with cations like Al^3+^, Fe^3+^ and Ca^2+^ and tends to firmly absorbs onto clay minerals and metallic oxides (Walter et al., 2000; Bowler et al., 2010; Abel, 2017). Moreover, we have used the positive extraction mode to identify the different minerals and elements. (see Supporting Information). K-related, Si-related and Na-related minerals were easily distinguished by PCA. We show that, the positive extraction mode is complementary to EDX analysis and the topography resulted by water-jet cutting has minor influence on data acquisition. Compared to EDX, ToF-SIMS has a better sensitivity and spatial resolution, thereby combination of both positive and negative extraction modes in ToF-SIMS analysis can be used to study rhizosphere PO_*x*_^–^ distribution and bioavailability of P at various stress conditions.

#### SEM-BSE and SEM-EDX

**FIGURE 5:**
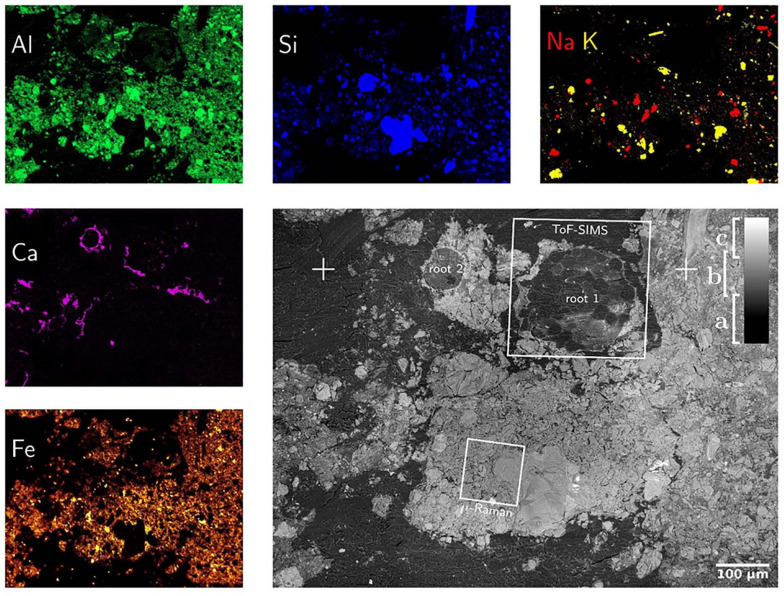
SEM-BSE (gray figure) and SEM-EDX maps of mineral-forming elements. SEM-BSE contrast allows for separation of resin (a), light (b) and heavy (c) minerals as well as the identification of root-cells. The positions marked as “ + ” are identical with those in Figure 3. The SEM-EDX false-color maps show exactly the same field-of-view such that SEM-BSE contrast can be linked to elemental composition. Please note that the Calcium distribution is almost identical to that of phosphorous which hints to calcium phosphates precipitated around the root surface.

Both, SEM-BSE and SEM-EDX maps provide chemical information about the surface of soil-disk. Electrons back-scatter more efficiently on heavy than on light elements, such that SEM-BSE contrast is used to distinguish regions of low electron density, resin and soil-organic matter, from electron-dense ones, namely mineral particles as shown in Figure 5. In this way mineral classes of different electron density are distinguishable, for instance sodium-feldspar and quartz from iron-minerals. In addition, element-distribution maps were measured by SEM-EDX. In principle any element heavier than berryllium can be measured by SEM-EDX with increasing sensitivity for heavier elements. Major element mapping was feasible starting with C since, the concentration of B was too low to be detected with SEM-EDX. Both, SEM-BSE and SEM-EDX micrographs proved a good overview on the soil composition (Figure 5). Mainly Si, Na, and Al containing minerals, e.g., quartz and Na-feldspar, were identified but also Ca, P and Fe are detected. At the root wall Ca precipitation is noticed. This may be due to the nutrient uptake regulation mechanisms by the plant root or the changes in pH (Walter et al., 2000, 2003; Shomer et al., 2003; Bowler et al., 2010; Abel, 2017; Byrt et al., 2018). As LR white is composed of C, H, and O, the infiltrated resin is clearly identified as C in SEM-EDX maps. Using SEM-BSE, resin can be separated from the soil matrix as shown in Figure 5. It should be noted that LR white contains traces of Na, Al, Si, S, and Cl (see Supporting Information) which must not falsely be interpreted as minerals. Also, the separation and identification of soil organic matter by SEM-EDX is challenging once embedded in LR white. Nevertheless, the correlation of HIM, SEM-BSE and SEM-EDX is an ideal tool to characterize morphology and elemental composition of minerals in soil (Hapca et al., 2011, 2015).

#### NanoSIMS

**FIGURE 6:**
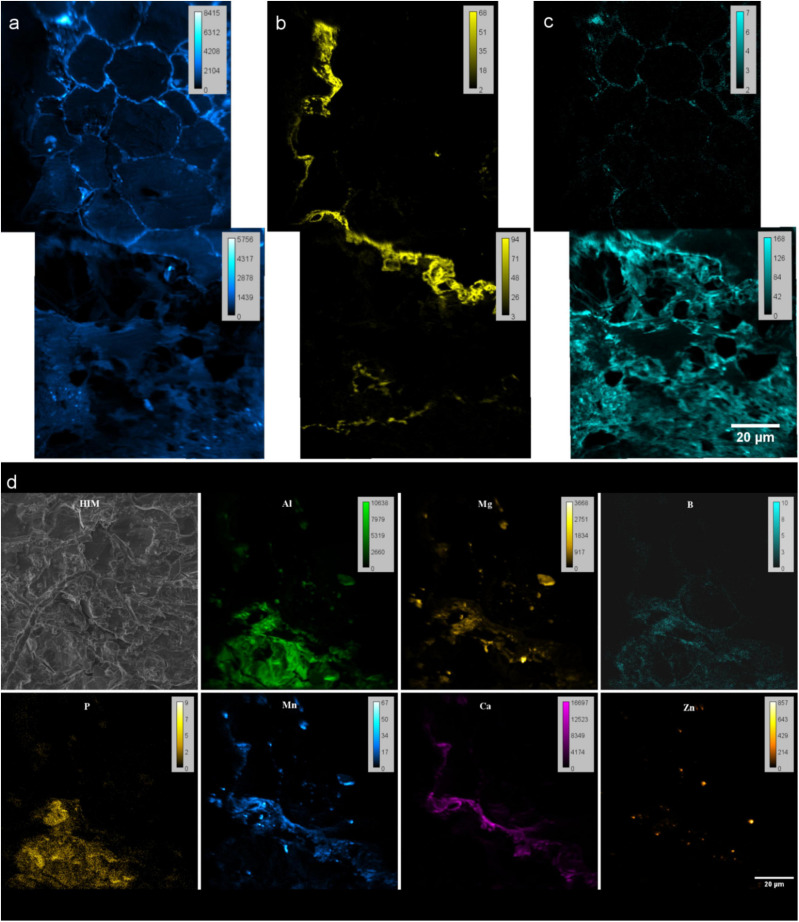
NanoSIMS images of resin embedded Rhizosphere. **(a–c)** Spectra from negative extraction mode showing elemental distribution **(a)** ^12^C^14^N^−^ **(b)** ^31^P^16^O_x_^−^ and **(c)** ^32^S^−^ at root-soil interface of rhizosphere. Two FoV’s stitched by MosaicJ in Fiji. **(d)** Maps from positive extraction mode showing elemental distributions of ^27^Al^+^, ^24^Mg^+^, ^11^B^+^, ^31^P^+^, ^55^Mn^+^, ^40^Ca^+^ and ^64^Zn^+^. **(d)** Helium ion micrograph (HIM) of the analyzed area.

NanoSIMS negative mode imaging shows the distribution of ^12^C^14^N^–^, ^31^P^16^O_*x*_^–^ and ^32^S^–^ secondary ions in the embedded rhizosphere sample. The ^12^C^14^N^–^ helped to differentiate the organic matter from the resin (Figures 6a–c). Therefore, it shows the cell arrangement of the root. ^31^P^16^O_*x*_^–^ was mainly found at the outer root wall and extend to the soil matrix. At the side where ^31^P is precipitated on the root epidermis, ^31^P was not abundant in the cortex cell walls. On the side where ^31^P is not precipitated, ^31^P is distributed within cortex cell walls of the root (see Supporting Information). ^32^S^–^ was detected on cortex cell walls.

With the positive extraction mode, we have identified the distribution of ^27^Al^+^, ^24^Mg^+^, ^11^B^+^, ^31^P^+^, ^55^Mn^+^, ^40^Ca^+^ and ^64^Zn^+^ (Figure 6d). Though boron could not be identified by EDX, capability of detection with nanoSIMS allowed to study B in rhizosphere as an important micronutrient for the quality and yields of crops, and a cell wall component.

Combination of positive and negative extraction modes showed distribution patterns of PO_*x*_^–^, highly correlates with cations Ca^2+^ and Mn^2+^ and some to extent of Mg^2+^ and Al^3 +^ at the root’s outer surface (Figures 6b,d-Ca). This can be related to the co-precipitation of P as highly insoluble Ca, and Al salts reducing the bio-availability of P for plants (Walter et al., 2000, 2003; Bowler et al., 2010; Byrt et al., 2018). Possibility of detecting ^12^C^14^N^–^,^31^P^16^O_*x*_^–^, ^32^S^–^, ^27^Al^+^, ^24^Mg^+^, ^11^B^+^, ^31^P^+^, ^55^Mn^+^, ^40^Ca^2+^ and ^64^Zn^2+^ allow to study the micro nutrient related research and their pathways within rhizosphere coupled to various correlative techniques.

#### μ-Raman

**FIGURE 7:**
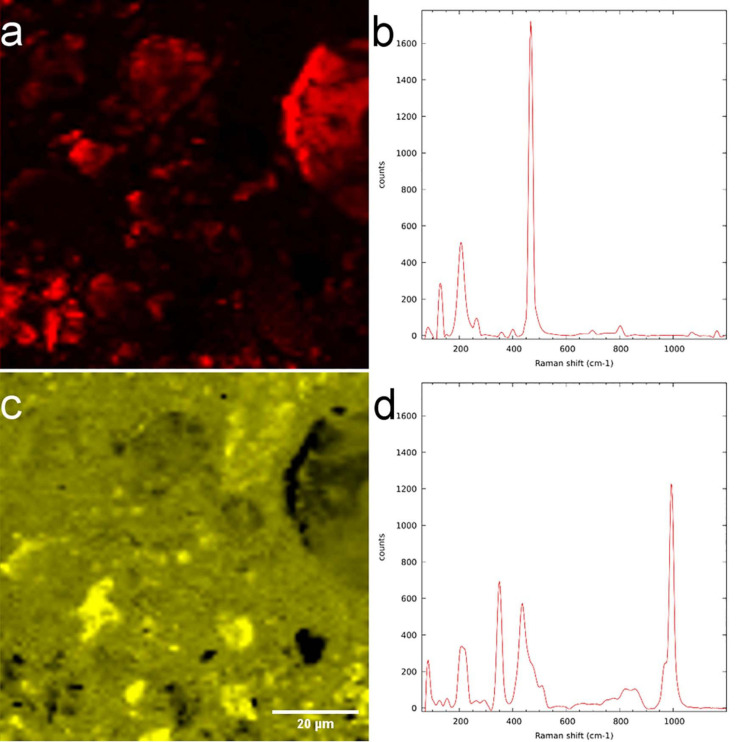
μ-Raman measurement of LR white resin-embedded soil. **(a)** Quartz-distribution map and **(b)** spectrum. **(c)** Mixture of zircon and aluminum-containing minerals and their **(d)** spectrum.

Employing WiTEC’s True Component analysis^®^ after μ-Raman mapping we could distinguish quartz minerals from Al-rich feldspar minerals similar to SEM-EDX. Figure 7a shows the map of quartz and its spectrum (Figure 7b), with the prominent peak at 468 cm^–1^ corresponding to Si-O-Si stretching. Figures 7c,d shows an Al-containing mineral and its spectrum, respectively, identified in the same field-of-view. Here μ-Raman adds additional information to SEM-EDX by revealing that the spectrum indicating measured minerals are a mixture of two minerals, which consists of 59.52% zircon and 40.48% of ammonium and sulfate containing minerals rather than a single pure component (Figure 7d), whereas SEM only provided elemental information (see Supporting Information). The dual peaks at 350 cm^–1^ and 436 cm^–1^ and peak with a shoulder at 1000 cm^–1^ may corresponds to zircon while the sharp peak at 995 cm^–1^ as well as two smaller yet broader peaks at 450 cm^–1^ and 600 cm^–1^ correspond to ammonium sulfate (see Supporting Table 2). Possible source would be aluminum sulfate and ammonium nitrate which were supplied as nutrients during the soil column preparation (Vetterlein et al., 2020). The Raman spectra of LR white resin has a sharp peak with two shoulders at 2950 cm^–1^ corresponding to C-H stretching and a small sharp peak at 1450 cm^–1^ corresponding to C = O (see Supporting Information). Therefore, using Raman spectroscopy we could accurately identify interested minerals in the resin imbedded sample, but not the biomolecules.

### The Spatial Distribution of Bacteria and CARD-FISH

**FIGURE 8:**
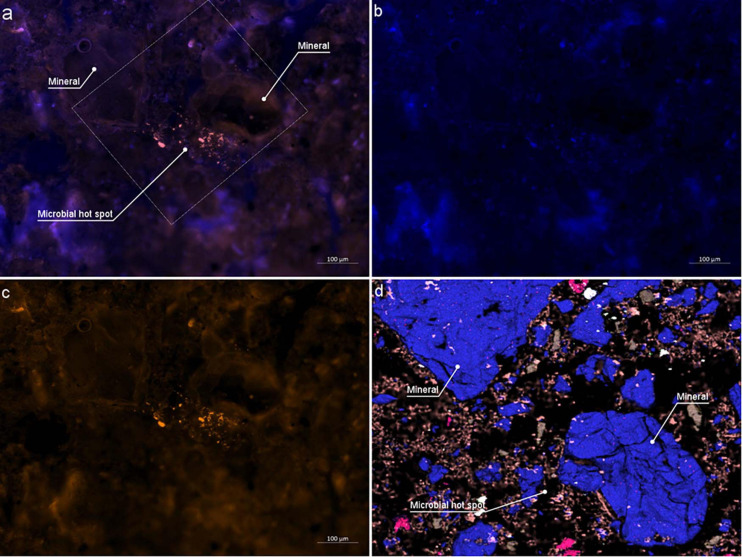
Catalyzed Reporter Deposition-Fluorescence *in situ* Hybridization (CARD-FISH) stained bacteria colonizing on Rhizosphere mineral. **(a)** Epifluorescence micrograph of combined DAPI and DsRed channel. Outlined square shows the area investigated by EDX analysis **(b)** DAPI channel **(c)** DsRed channel **(d)** Same area imaged by EDX. Two larger minerals indicate Si rich minerals where bacterial aggregates are identified.

On the LR white embedded sample, we could successfully identify bacterial aggregates within the soil using CARD-FISH (Figure 8). Bacteria fluoresced in DsRed channel (Figure 8c) is false colored in red orange under excitation wavelength of 590 nm after successful hybridization, making it easy to distinguish bacteria from the native autofluorescence of the soil (see Supporting Figure 8 for similar results). In contrast, the DAPI staining was non-specifically binding to soil components and resin, making difficult cell identification (Figure 8b). However, for each positively hybridized microbial cell, the DAPI signal was verified to coincide with the Alexa594 signal (Figure 8a). Bacteria were often observed as clusters or agglomerations rarely in the vicinity of root sections and mostly in the vicinity of quartz minerals (Figure 8d). As stated in the literature (Nunan et al., 2001; Klauth et al., 2004), it is evident that nuclear stains are not capable of identifying bacteria in soil and specialized CARD-FISH methods are required for a precise identification. However, single microbial cells are hard to visualize due to high background fluorescence, particularly of the roots and associated minerals, we therefore cannot exclude the presence of these in the root surroundings. Correlative EDX analysis (Figure 8d) shows the elemental composition of the minerals in the vicinity of the microbial hot spots. However, further investigations are necessary to determine the type of minerals to which the bacteria colonize.

The success of the CARD-FISH experiment strongly depends on the temperature at which the resin is cured. Therefore, we cured the resin at 48°C, and at 60°C to test the hybridization success. CARD-FISH were only successful at 48°C, which is well below the standard polymerization temperature of 60°C. Polymerization of LR white is an exothermic reaction and curing at 60°C can further increase the embedding temperature leading to potential degradation of the bacterial ribosomes, also have a negative effect of probe permeability through the strongly polymerized resin. These results are in-line with our observations, where CARD-FISH experiments were not successful with the samples cured at 60°C, instead hybridization was observed only at 48°C. Thus, if correlation with CARD-FISH is desired, temperature of 48°C should be used for curing and found to be compatible for high vacuum microscopy.

Different microscopy techniques may alter the surface topography due to beam-sample interactions and lead to artifacts. (Supporting Figure 6) As such, it is important to setup the characterization cascade starting with techniques that cause minimum damages while measurement. This will allow to acquire the maximum set of correlative data with minimum artifacts. Here, we suggest a characterization cascade starting with light microscopy for mapping, followed by CARD-FISH and epifluorescence microscopy. Then the following order: HIM → ToF-SIMS → SEM/EDX → nanoSIMS →μ-Raman for high-resolution characterization. HIM and ToF-SIMS being surface sensitive techniques, should be completed at earlier stages. Starting from SEM, sample made conductive by depositing a conductive metal layer, leading to minor surface modifications. NanoSIMS is a destructive technique providing hard ionization of sample material up to single atomic ions for analysis of the isotopic composition resulting major pits in the sample. Hence it is to be carried out after SEM/EDX. Raman scanning is the last as it is time consuming and the laser can cause the resin evaporation, but minerals are unlikely to be altered by other techniques.

### Image Registration With the Correlia Plugin in Fiji

**FIGURE 9:**
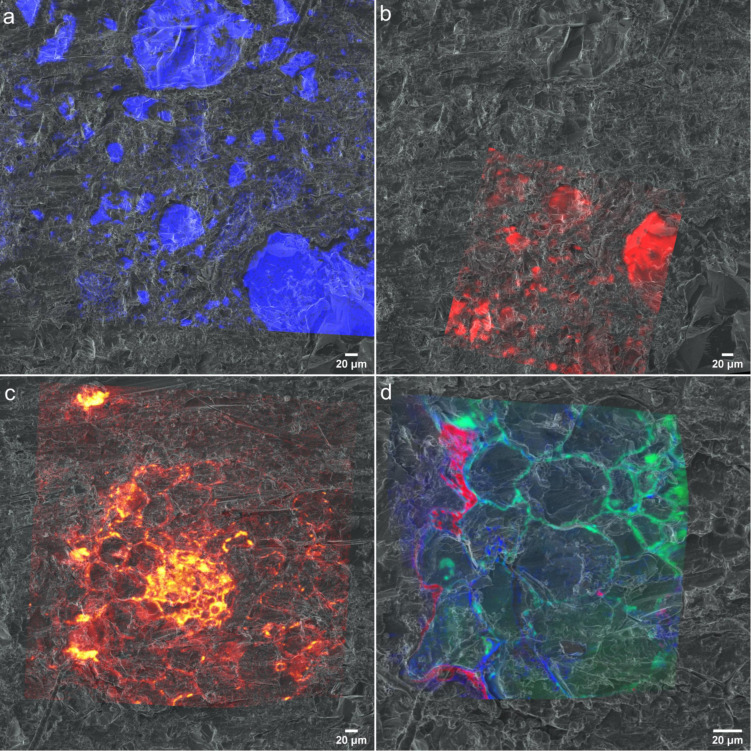
Chemical maps registered to helium ion micrographs **(a)** EDX spectra of Si registered onto HIM **(b)** Raman spectra resisted on to HIM **(c)** CNO^–^ spectra of ToF-SIMS registered onto HIM. **(d)** ^12^C^14^N^−^, ^31^P^−^ and ^32^S^−^ distribution maps from nanoSIMS registered onto HIM.

For a mechanistic understanding of complex patterns of interactions between soil, microbiome and the roots using a correlative approach, multiple images acquired by various techniques needs to be successfully registered and to be analyzed. For the image registration we have used helium ion micrographs as the base image to register all high-resolution chemical micrographs as the first step as similar features can be recognized. Then HIM was registered on to the optical micrograph based on common surface features. (Supporting Figure 7) HIM is an excellent alternative to optical or SEM micrographs as it can be used as early characterization tool in this workflow providing excellent resolution and quick image acquisition compared to other techniques. Figure 9 demonstrates the registration of (a) SEM-EDX, (b) μ-Raman, (c) ToF-SIMS, and (d) nanoSIMS micrographs onto HIM micrograph. Our results demonstrate HIM is a well-suited for non-quantitative imaging of the embedded rhizosphere sample to prepare treasure maps of the interested RoI and image registration. However, in a correlative workflow involving X-Ray microCT, BSE is a promising candidate for 2D-3D image registrations due to their similar contrast.

### Validation and Application of the Method

Here we presented six analytical instruments that are compatible with our embedding method. This is an indication of the compatibility of our methodology for a wide variety of analytical tools which can be used in combination for root-soil-microbe characterization. Though multiple instrumentation will help acquiring a concise dataset, it is not mandatory to include all of these techniques for a conclusive outcome for a particular research question. Therefore, these analytical techniques can be used in different combinations to address particular research question accordingly.

**FIGURE 10:**
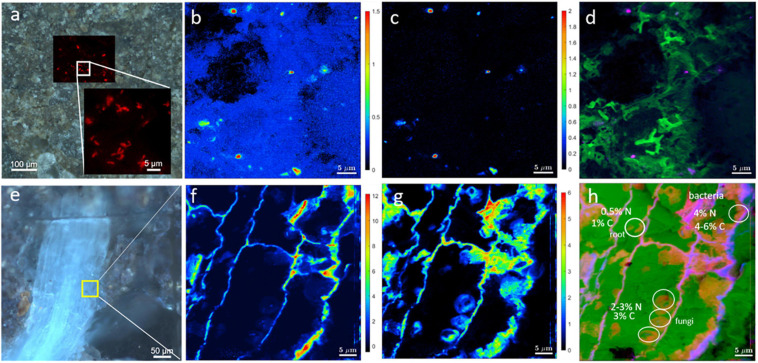
Correlation of light microscopy with SIP-nanoSIMS showing C and N uptake by roots and bacteria. **(a)** CARD-FISH image of soil microbes overlaid with brightfield micrograph showing the location of microbial cluster in the resin-embedded rhizosphere sample. The inset in frame a depicts an enlarged CARD-FISH image of bacterial cluster. NanoSIMS imaging of soil bacteria **(b–d)** and root **(f–h)**. NanoSIMS analysis shows the relative assimilation of carbon **(b)** and nitrogen **(c)** within microbial cluster. **(d)** Log-scaled RGB-overlay of nitrogen assimilation (Red channel), CN^–^ ion yield map as biomass intrinsic marker (Green channel) and carbon assimilation (Blue channel). **(e)** Epifluorescence image showing the root (blue) and adjacent minerals. The area analyzed by nanoSIMS is highlighted in yellow. Relative assimilation of carbon **(f)** and nitrogen **(g)** in the root. **(h)** Log-scaled RGB-overlay of assimilation activity (nitrogen in Red, carbon in Blue) with the intrinsic biomass marker (CN^–^ ion yield map in Green).

As an example, the mere correlation of CARD-FISH with Stable Isotope Probing (SIP)-nanoSIMS is demonstrated in Figures 10a–d. First, we had located a microbial cluster in soil by the CARD-FISH and then used nanoSIMS to determine C (Figure 10b) and N (Figure 10c) assimilation. The root is enriched in both, ^13^C and ^15^N, (Figures 10e–h), whereas bacteria located in soil are not enriched in ^13^C, indicating that this particular bacterial cluster had not accessed plant organic matter during the time of the experiment. Light micrographs (Figures 10a,e) and nanoSIMS images are complimentary and validate the correct identification of soil bacteria and determine bacterial distributions within rhizosphere. This type of analysis will help understand the microbial contributions for nutrient cycles at scales relevant to microbes.

By correlating CARD-FISH result with distribution maps of chemical elements or minerals measured by EDX and μ-Raman, respectively, the microenvironment of the microbial cluster can be studied, as shown in Figure 8d. If statistically analyzed this would allow for drawing conclusions to which minerals certain bacteria preferentially attached. These different correlations demonstrate the technical advancements added to soil research when combining root-bacteria-soil characterization which is made possible by this new embedding method. For a statistical conclusion, further studies are necessary, which are beyond the scope of this work.

## Limitations, Challenges and Outlook of the Method

Our method is optimized for the Haplic Phaeozem soil, however, the reported experimental conditions can be used as a starting point for the embedding of other types of soil. As the physical, chemical, and water retaining properties of different soils are significantly different from each other, the dehydration step may need to be adjusted accordingly for a particular soil type. Apart from loamy soil, we have embedded sandy soil with the same conditions, and obtained similar embedding quality. (see Supporting Figure 10, 12) Care needs to be taken during the curing step to maintain anaerobic conditions as LR white resin is highly sensitive to oxygen. We have carried out embedding with five samples per batch, in duplicate, to test our methodology. All ten samples were embedded successfully, indicating the reproducibility of this technique (data not shown).

Soil autofluorescence is a challenge for μ-RAMAN characterization. A way to suppress it, is to use long wavelength lasers (red or IR), however, this will in turn lower the lateral resolution of the technique. Laser power and wavelength must be selected according to the targeted compounds of interest in the sample. For minerals, high power can be used, but will result in degeneration of the resin.

Rotation of the sample during mounting into multiple instruments is taking place at the current setup. As such acquired data show a different orientation. Once registered on a single plane, these slight rotations become visible (Supporting Figure 9a). This limits the total common area for data analysis with all instruments. This requires to acquire data from larger areas than necessary to minimize loss of correlative data during the analysis. This is to be mitigated by using a single sample holder with a defined coordinate system which has adapters to fit sample into different instruments. This will allow to maintain the same orientation in multiple platforms, thereby multiple data from different instruments has the same orientation, and thereby larger portion of acquired data can be correlated.

In nanoSIMS, data acquisition area is less than 100 × 100 μm, which is the smallest field of data acquisition. Accuracy of sample maneuvering to exact position is critical to obtain correlative data. Provided the sample is 16 mm in diameter, and instruments limitations in optics, it is time consuming to relocate to the analysis position with an accuracy of 50 μm. Hence a prerecorded coordinate system with a marking on the surface will help to identify RoIs for nanoSIMS, and pre-image registration using secondary electron image will save lots of analytical time.

Catalyzed Reporter Deposition-Fluorescence *in situ* Hybridization is essential for specific identification, quantification and visualization of microorganisms. By integrating this approach in to the current workflow, phylogenetic information is linked to morphology, structure and cellular activity in a spatial context. Moreover, by using specifically design sequence-specific probes, phylotypes of interest can be traced and characterized.

## Conclusion

Here we presented a new embedding method that allows for a comprehensive correlative microscopic and spectrometry approach which paves the way for a synergistic understanding of complex soil-plant-microbe interactions via multiple microscopic and spectrometry techniques. Introduced LR white embedding and waterjet cutting showed capability of preparing the rhizosphere soil matrix compatible for high-resolution chemical microscopy and identification of bacteria by CARD-FISH, which were not combined before in a correlative approach. Now vital research studies can be carried out using a variety of instruments with special analytical capabilities, covering all components of rhizosphere simultaneously: soil-root-microbes. Overall, this method allows to comprehensively study the spatio-temporal organization of nutrients and microbes in the rhizosphere at μm to nm scale and we hope this will make a platform for mechanistic understanding of complex patterns of interactions between roots, microbiome and the soil in a correlative microscopy approach in the future.

## Data Availability Statement

The raw data supporting the conclusions of this article will be made available by the authors, without undue reservation. Data set is provided through the link: http://www.ufz.de/record/dmp/archive/11158/de/.

## Author Contributions

CB developed the embedding method, HIM imaging, epifluorescence imaging, ToF-SIMS analysis, image registration, data analysis and wrote the initial draft. YD analyzed the Raman data and contributed to manuscript editing. MS did the HIM imaging, Raman and SEM/EDX analysis, image registration and contributed to manuscript writing. HS did the ToF-SIMS and nanoSIMS experiments and data analysis and contributed to manuscript writing. HR acquired funding, managed the overall project, contributed to manuscript editing and project supervision. NM did the CARD-FISH experiments, project supervision and contributed to manuscript writing. All authors read and agreed on the final version of the manuscript.

## Conflict of Interest

The authors declare that the research was conducted in the absence of any commercial or financial relationships that could be construed as a potential conflict of interest.
