# Corrigendum: Microbial identification, high-resolution microscopy and spectrometry of the rhizosphere in its native spatial context

**DOI:** 10.3389/fpls.2022.1125001

**Published:** 2023-01-09

**Authors:** Chaturanga D. Bandara, Matthias Schmidt, Yalda Davoudpour, Hryhoriy Stryhanyuk, Hans H. Richnow, Niculina Musat

**Affiliations:** ProVIS-Centre for Chemical Microscopy, Department of Isotope Biogeochemistry, Helmholtz Center for Environmental Research (UFZ), Leipzig, Germany

**Keywords:** CARD-FISH, correlative chemical microscopy, helium ion microscopy, London resin white embedding, rhizosphere, secondary ion mass spectrometry, soil bacteria, water-jet cutting

In the published article the Funding Statement was missing. The Funding Statement appears below.

