# Supplementary material for: Microbial Identification, High-Resolution Microscopy and Spectrometry of the Rhizosphere in Its Native Spatial Context
Source: Front Plant Sci. 2021 Jul 7;12:668929. doi: 10.3389/fpls.2021.668929 (PMC8293618; doi:10.3389/fpls.2021.668929)
Supplement: Supplementary file 1 [file Data_Sheet_1.docx]

**Supporting Information**

Microbial Identification, High-Resolution Microscopy and Spectrometry of the Rhizosphere in its Native Spatial Context

Running Title: Correlative microscopy of the rhizosphere

Chaturanga D. Bandara*, Matthias Schmidt, Yalda Davoudpour, Hryhoriy Stryhanyuk*, Hans H. Richnow, Niculina Musat

ProVIS-Centre for Correlative Microscopy, Department of Isotope Biogeochemistry, Helmholtz-Center for Environmental Research (UFZ), Permoserstraße15, 04318 Leipzig, Germany

Corresponding authors: chatu.im@ufz.de, gregory.stryhanyuk@ufz.de

## Application of multiple micro analytical tools in soil research

***HIM*** is an extremely surface-sensitive (probing only a few nm below the surface), high-resolution (down to 0.25nm) microscopy technique with numerous applications in the life-sciences. ^1-3^ The technique allows for investigating insulating samples owing to efficient charge-compensation with an electron flood-gun, ^1, 4^ On top of that, HIM imaging is operating at beam-currents in the sub-pico-amp range which in turn means that only a few tens of ions are being implanted per pixel during image acquisition causing only a minimum damage to the sample.^2-3, 5^ To the best of our knowledge, to-date only one publication in soil research exists where HIM was used to study mineral-organic interaction. ^3^ Therefore, adding HIM to the toolbox of correlative analysis of the rhizosphere with high-resolution 2D imaging techniques provides additional insight to soil biochemical processes at nanoscale of intact rhizosphere.

***SEM*** is a well-established surface-analytical technique in soil-science and geology. Whilst the commonly used secondary electron (SE) imaging provides high lateral resolution and surface sensitivity no chemical information about the imaged material can be gained. In contrast, a material contrast increasing with an electron density is obtained when using electron acceleration energies of >=10 kV and detecting back-scattered electrons (BSE).^5^ In resin-embedded soil this allows for separating resin and organic matter from mineral particles. Furthermore, element-specific chemical information of the soil sample, in particular to identify mineral particles, can be obtained when the SEM is combined with an EDX.^6^

***ToF-SIMS*** allows for studying the molecular composition of heterogeneous organic/inorganic samples with about 100 nm spatial resolution and simultaneous detection of molecular fragments with mass-resolving power >5000 (MRP=dM/M) in a broad mass range up to ~10 kDa.^7-8^ The application of cluster-ion sources in a ToF-SIMS experiment reduces molecular fragmentation and allows for high-resolution 3D analysis of organics without having to destroy the spatial organization of the sample by chemical extraction. This renders ToF-SIMS a potential technique to study biomarkers in complex mineral-organic soil specimens.^9-12^ Recent trend in advances of complex ToF-SIMS data analysis including Principal component analysis (PCA), multivariate curve resolution-altering least squares (MCR-ALS), G-SIMS^13^ and artificial intelligence methods like neural networks and novel informatics-based methods^11, 14-20^ will eventually lead to identify various molecules in a complex mixture of organics in rhizosphere sample in the future.

***nanoSIMS*** allows for elemental/isotope-resolved mapping of sample surface with simultaneous detection of ion counts for up to 7 isotopes (nanoSIMS 50L), lateral resolution down to 50 nm and mass-resolving power (MRP=M/dM) above 8000.^21^ Combining nanoSIMS with Fluorescence and or Halogen *In-situ* Hybridization (FISH/HISH) and Stable Isotope Probing (SIP) allows for phenotypic identification of bacteria and quantitation of their metabolic activity in complex environmental samples.^22-24^ The recently commercialized RF-plasma source of O^-^ ions makes nanoSIMS feasible for high-resolution mapping of trace elements in biological systems.^25^

**µ-Raman** is based on the inelastic scattering of the monochromatic light at the surface of a sample and allows for spatially resolved molecular identification based on molecules unique vibrational characteristics.^26^ µ-Raman analysis does not require elaborative sample preparation steps which makes it easily applicable on a variety of samples from geological specimens to the characterization of soil, soil organic matter and carbon flows.^27-29^ Recent technical improvements extended its application in earth- and life-sciences, particularly for microbial cells analysis and isotopes.^30-31^ Thus, µ-Raman contributed to the identification and classification of soil bacteria and their metabolites in combination with stable isotope labelling and other single cell techniques such as namoSIMS^32-33^ e.g. spotting nitrogen fixing bacteria in soil community using ^15^N stable isotope labelling.^30, 34^


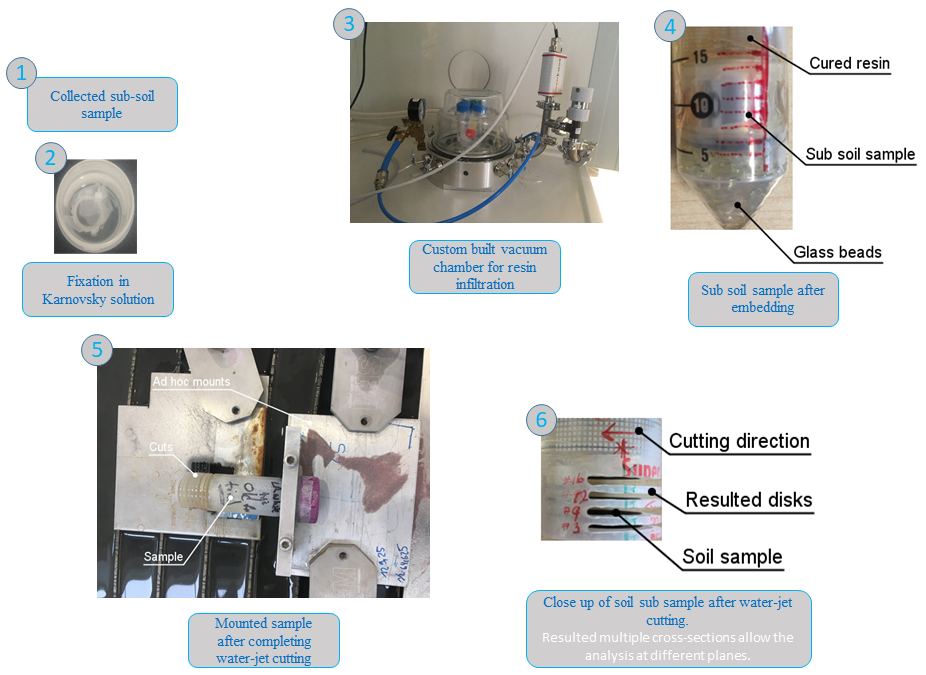


Supporting Figure S 1: Pictures from individual steps of the embedding method


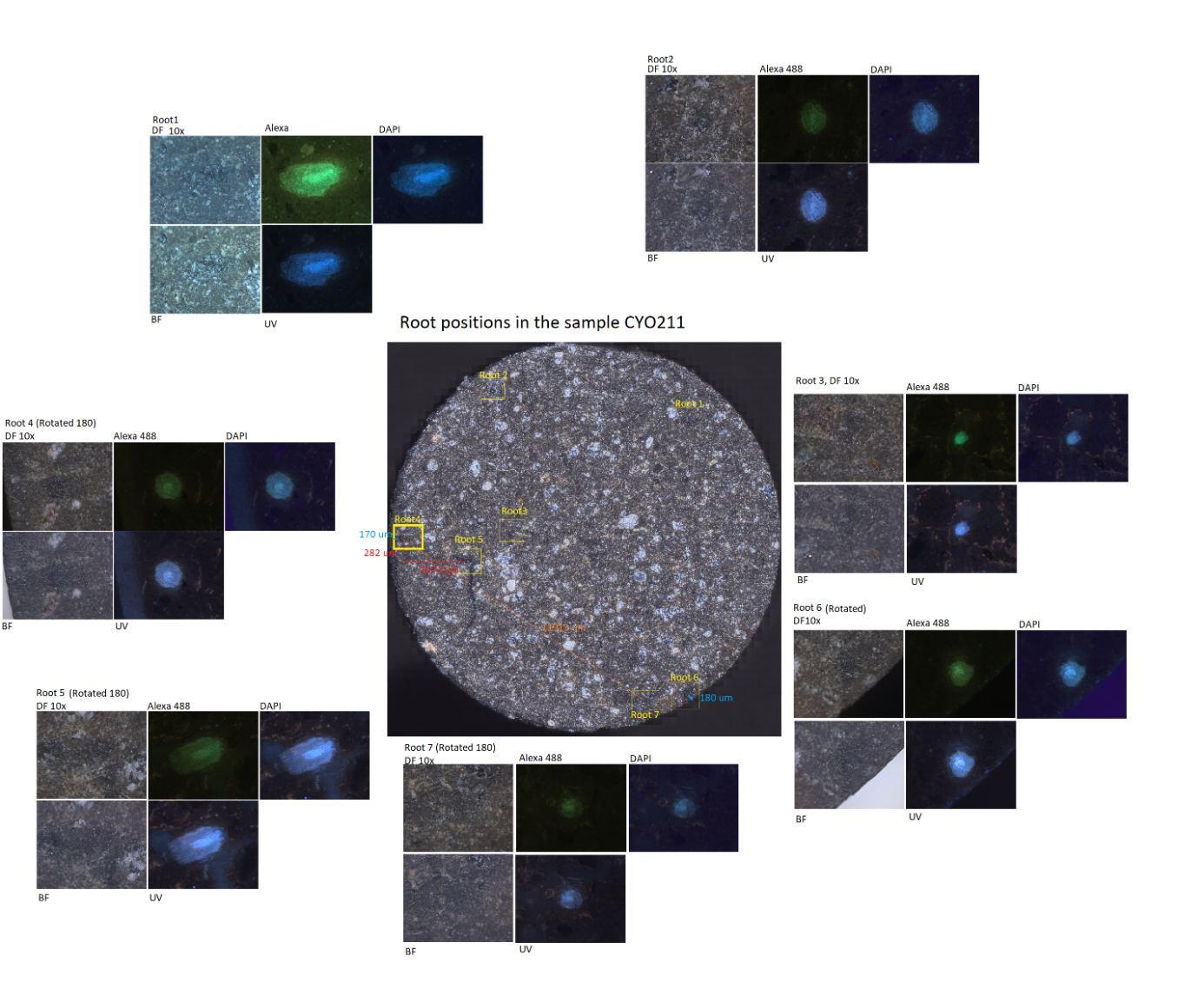


Supporting Figure S 2: Identification of RoI with Epifluorescence microscopy and prepared treasure map for high resolution microscopy

| Setting Number | Feed Speed in mm/min | Particle consumption rate g/min |
| --- | --- | --- |
| 1 | 54.7 | 400 |
| 2 | 54.7 | 400 |
| 3 | 27.35 | 400 |
| 4 | 5.55 | 400 |
| 5 | 125.9 | 350 |
| 6 | 89.2 | 350 |
| 7 | 65.3 | 500 |
| 8 | 32.6 | 500 |
| 9 | 6.63 | 500 |
| 10 | 220.8 | 350 |
| 11 | 111.4 | 350 |
| 12 | 25.8 | 400 |
| 13 | 12.9 | 400 |
| 14 | 16.9 | 400 |
| 15 | 14.7 | 450 |
| 16 | 7.3 | 450 |
| 17 | 169.7 | 350 |
| 18 | 84.8 | 350 |


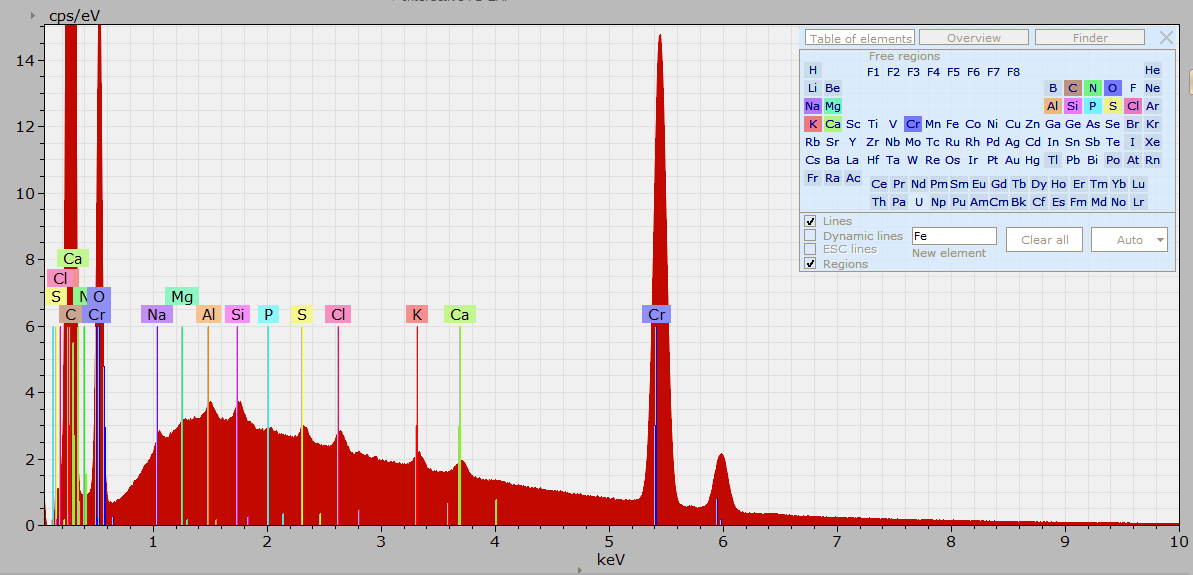


Supporting Figure S 3:EDX Spectra of LR white

Supporting Table 1: Water-jet cutting parameters operated at 3700 bar pressure


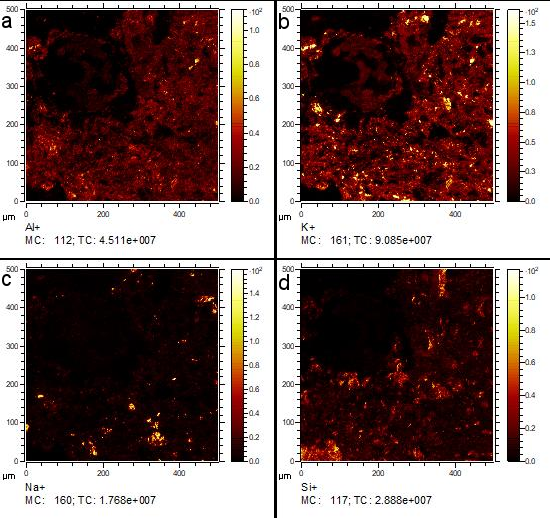


Supporting Figure S 4: ToF-SIMS of the rhizosphere in positive extraction mode. Al, K, Na and Si minerals are distinguished by Multivariate analysis. Root area shows no signal and hence dark.


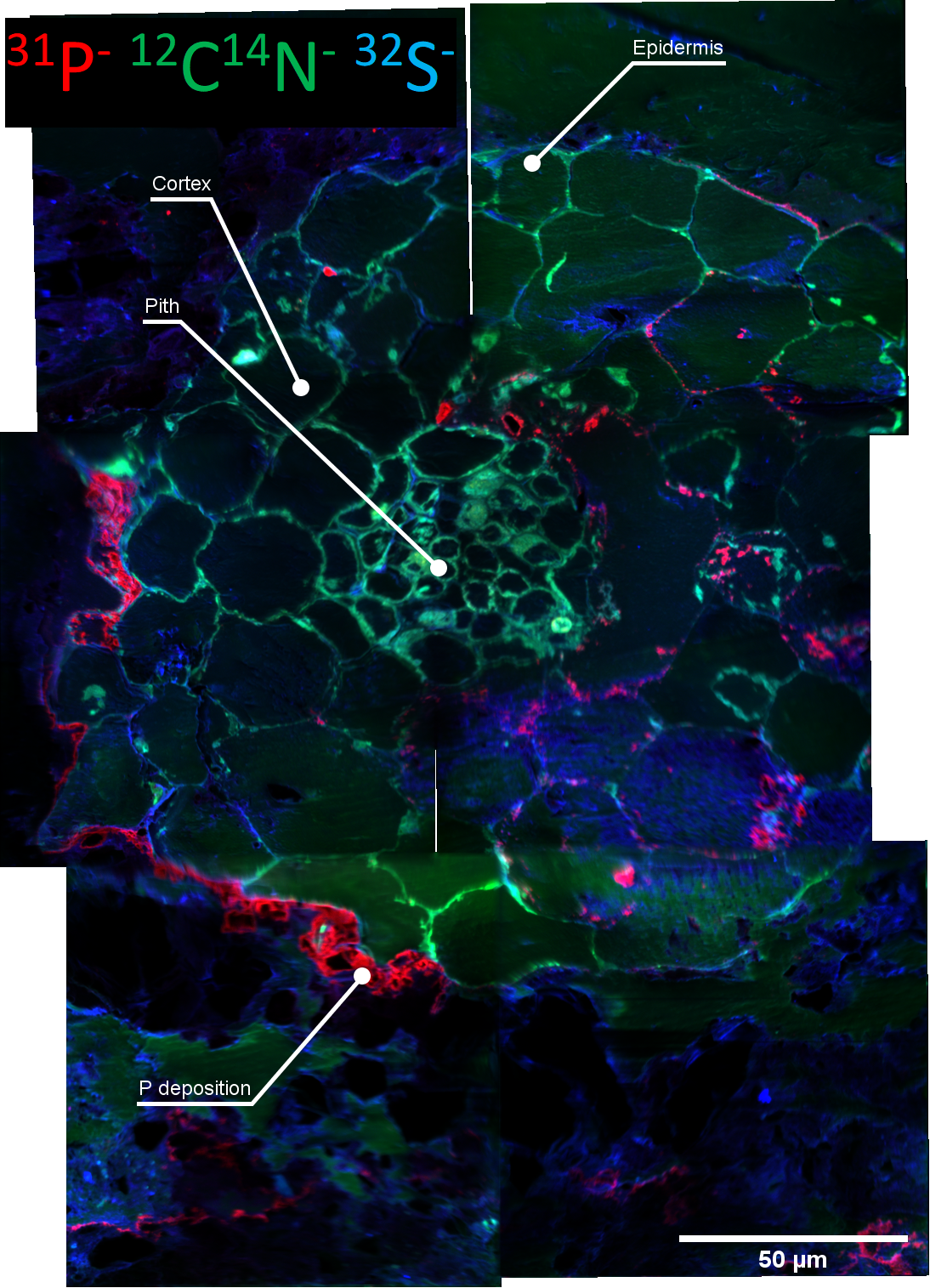


Supporting Figure S 5: nanoSIMS mosaic of rhizosphere showing ^31^P^-^, ^12^C^14^N^-^ and ^32^S^-^ distribution. Image is reconstructed by combining 7 individual FoVs. Individual image is 95 µm FOV. P deposition is prominent on the epidermis of bottom left quarter of the root. In the adjacent cortex P is not detected. When P is not detected on epidermis, it is detected on the adjacent cortex cells.

Supporting Table 2: Characteristic Raman peaks on Zircon and aluminium ammonium sulfate

| Sample | Band position (cm-1) | Suggested assignment |
| --- | --- | --- |
| Zircon^35^ | 1000  436  350 | Si-O stretching  Si-O bending  External mode |
|  |  |  |
| Aluminium ammonium slufate^36^ | 995 | SO_4_ |
|  | 600  450 | SO_4_, AlO_6_, Al-OH  Lattice modes of (AlO_6_), (Al-H_2_O), (SO_4_), (NH_4_) |


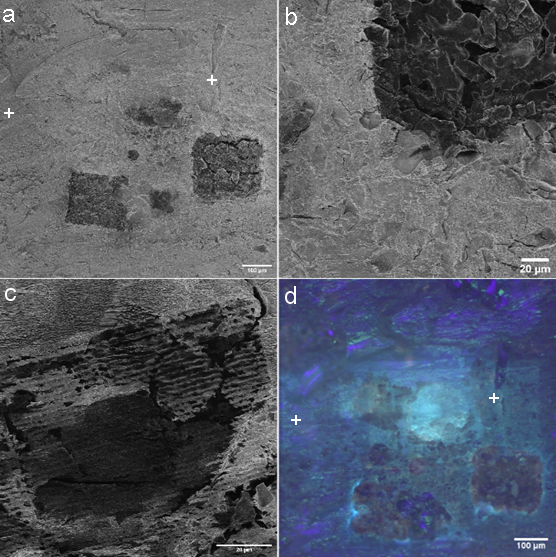


Supporting Figure S 6: Sample damage after correlative microscopy. a-c) HIM micrographs show the overview of the sample after multiple imaging. b) Damaged caused by 30mW power laser with 425 nm wavelength. c) Damaged caused by 10 mW power laser with 785 nm wavelength. Damage is much less compared to b. d) Epi-fluorescence micrograph show the difference in fluorescence in the sample after characterization by multiple microanalytical tools. Areas scanned with Raman microscope are not fluorescent.


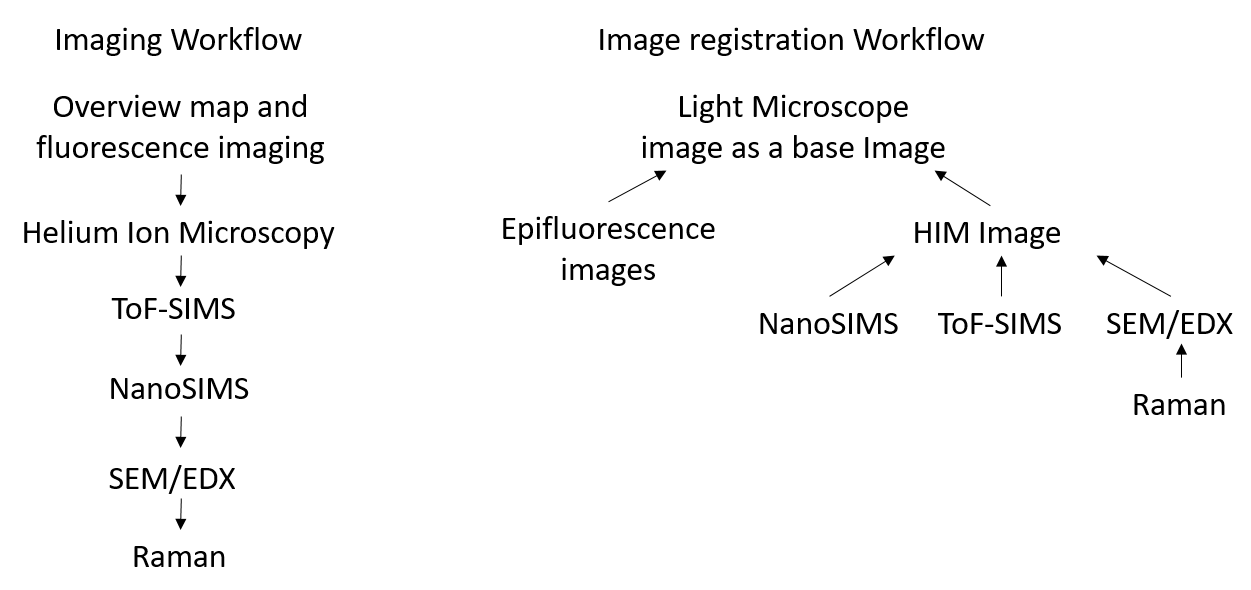


Supporting Figure S 7: Image acquisition and Image registration workflows. Imaging workflow shows the recommended image acquisition. Image registration suggests the high resolution images are to be first registered on to HIM, Raman images to the EDX map from SEM and then HIM image to be registered on to light micrographs.


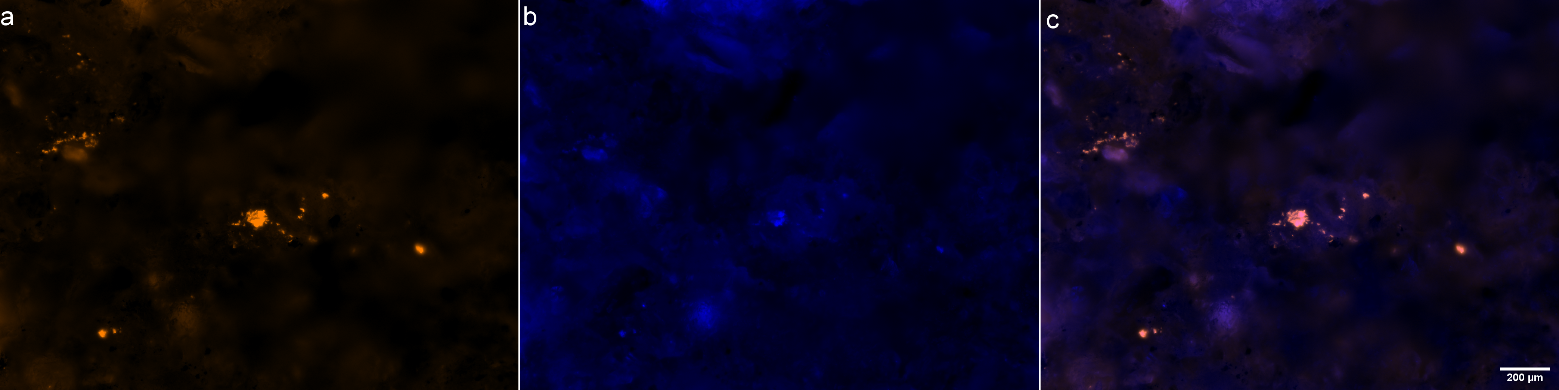


Supporting Figure S 8: CARD-FISH showing multiple bacterial colonies of embedded rhizosphere a) Ds Red b) DAPI c) Two channels combined


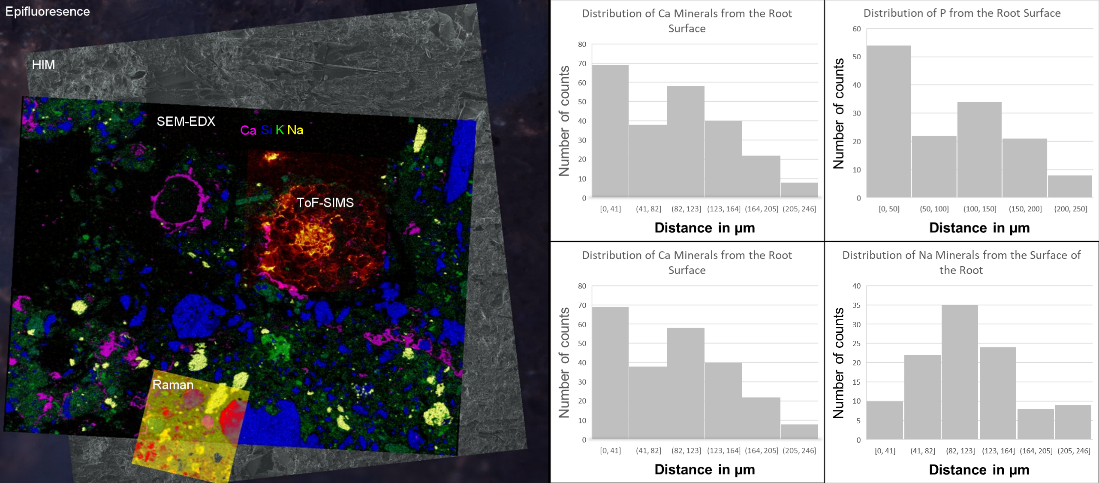


Supporting Figure S 9: Distribution of elements in the rhizosphere. Distance is calculated from the surface of the root.

Upon registration, different micrographs are oriented differently, there by, common area of overlap is reduced. Here, we demonstrate a possible analysis of registered EDX and ToF-SIMS data using the Correlia Plugin, showing the elemental distribution on a 2D plane from the epidermis of the root. As EDX cannot distinguish root cell wall due to the similar composition as of resin, use of ToF-SIMS could overcome the analysis difficulties, which is a highlight of correlation of multiple imaging techniques. This is only a plausible demonstration on various data analyses could be done with an availability of a method which can characterize the all-important components of the rhizosphere.


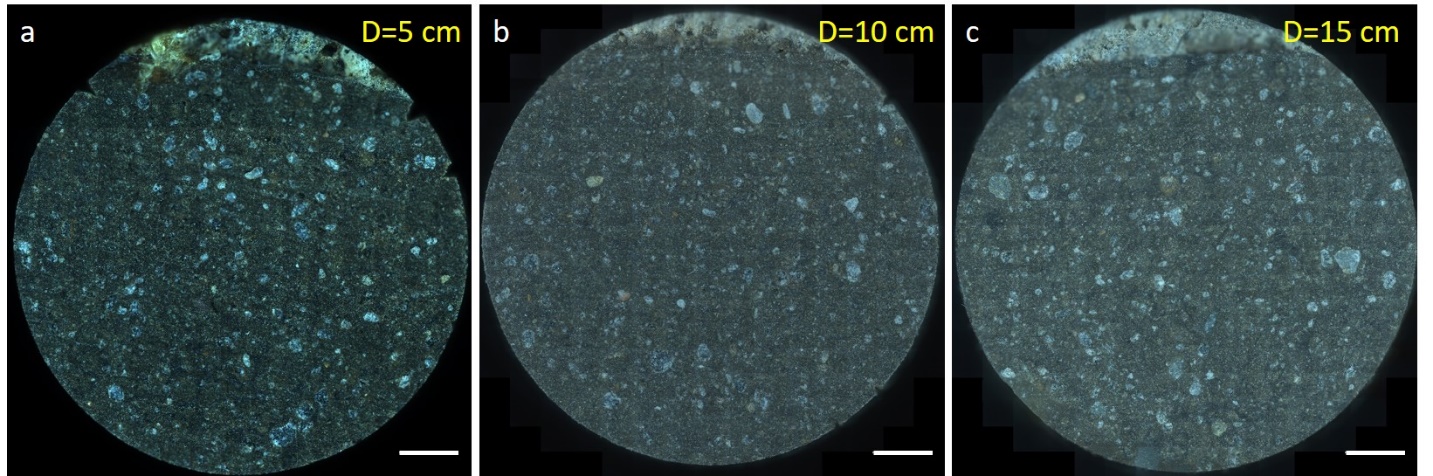


Supporting Figure S 10: Light micrograph mosaic of LR white embedded and waterjet cut soil sub-samples. Samples are acquired at a) Depth = 5 cm, b) Depth =10 cm, c) Depth= 15 cm were able to pre-process for microscopy with the same quality by our proposed method. Scale bar 2000 µm


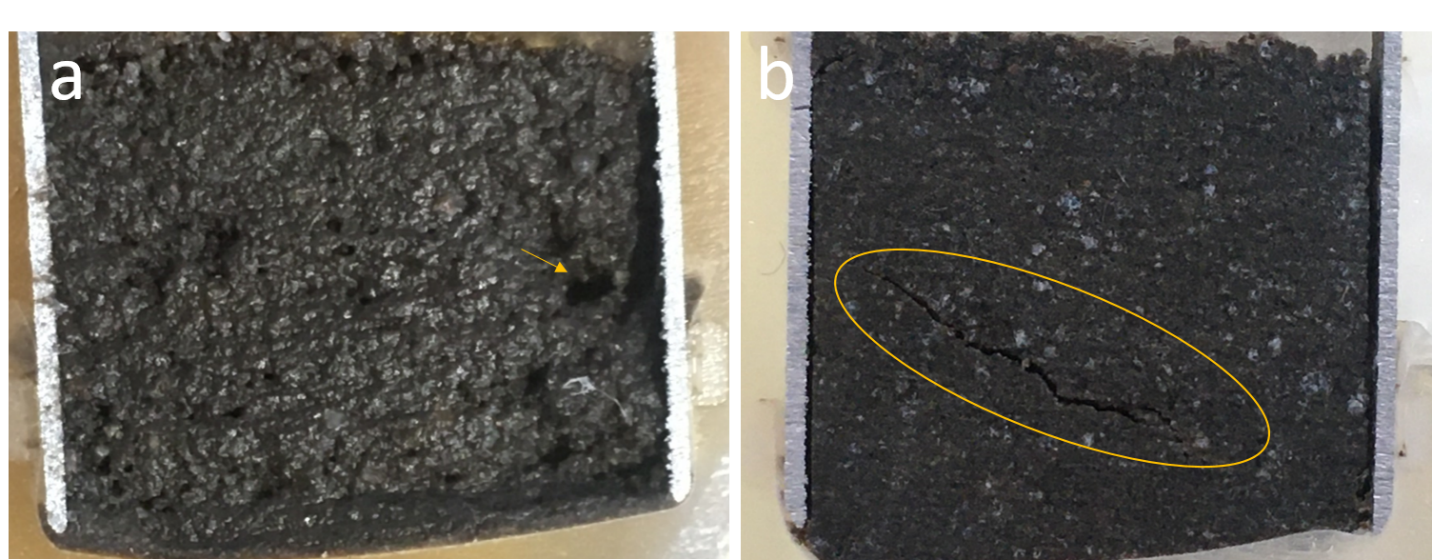


Supporting Figure S 11: Comparison of unsuccessful resin embedding attempts of a) Spurr resin and b) LR white thermally cured

For initial embedding quality tests samples were cross-sectioned in vertical direction. Resin embedding trials with spurr resin was not successful and often resulted in poor curing (Figure S11 a). After water-jet cutting sample surface was rough and had holes (indicated by a yellow arrow) due to uneven curing. Resulted sample surface was wet and sticky. Chemical cure of LR white was fast (2-3 minutes). However, cracks were formed within the sample (yellow circle) and in the resin reservoir outside Al cylinder (Figure S11 b). Sample was self-warmed due to exothermic polymerization reaction during chemical curing. Both spurr and LR white thermal curing approaches were inconsistent considering the quality of embedding. Therefore, these approaches were abandoned.


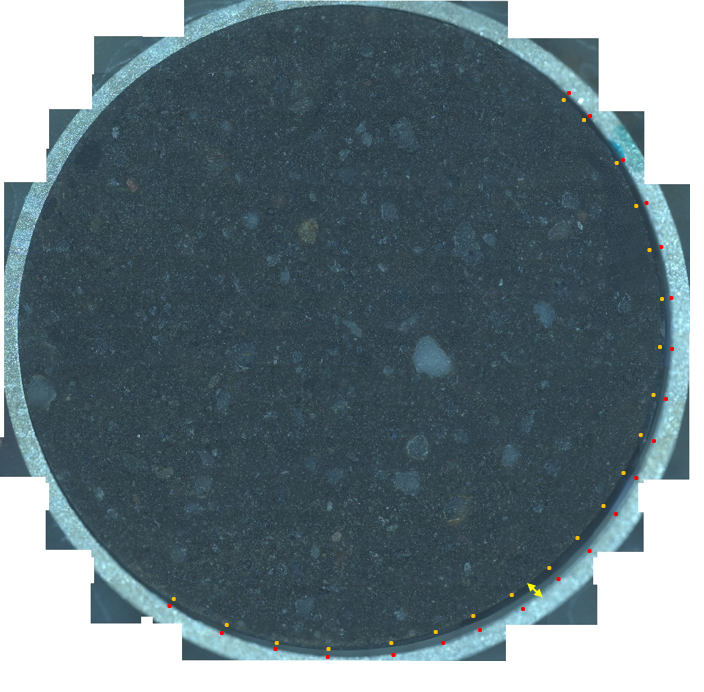


Supporting Figure S 12: Multiple darkfield micrographs show LR white embedded, sand soil disk. Double headed arrow shows the space between soil disk and the aluminium cylinder due to shrinkage of the soil disk. Yellow dots mark the border of soil disk, red dots mark inner wall of aluminum cylinder where separation is visible. However, sand soil show equal quality compared to loam soil with no visible cracks or non-solidified areas.

References

1. Ward, B. W.; Notte, J. A.; Economou, N. P., Helium ion microscope: A new tool for nanoscale microscopy and metrology. *Journal of Vacuum Science & Technology B: Microelectronics and Nanometer Structures* **2006,** *24* (6), 2871.

2. Bandara, C. D.; Ballerin, G.; Leppänen, M.; Tesfamichael, T.; Ostrikov, K.; Whitchurch, C. B., Resolving Bio-Nano Interactions of E.coli Bacteria-Dragonfly Wing Interface with Helium Ion and 3D-Structured Illumination Microscopy to Understand Bacterial Death on Nanotopography. *ACS Biomaterials Science & Engineering* **2020**.

3. Bandara, C. D.; Singh, S.; Afara, I. O.; Tesfamichael, T.; Wolff, A.; Ostrikov, K.; Oloyede, A., Bactericidal Effects of Natural Nanotopography of Dragonfly Wing on Escherichia coli. *ACS Applied Materials & Interfaces* **2017,** *9* (8), 6746-6760.

4. Joens, M. S.; Huynh, C.; Kasuboski, J. M.; Ferranti, D.; Sigal, Y. J.; Zeitvogel, F.; Obst, M.; Burkhardt, C. J.; Curran, K. P.; Chalasani, S. H.; Stern, L. A.; Goetze, B.; Fitzpatrick, J. A. J., Helium Ion Microscopy (HIM) for the imaging of biological samples at sub-nanometer resolution. *Sci. Rep.* **2013,** *3*, 3514.

5. Schmidt, M.; Byrne, J. M.; Maasilta, I. J., Bio-imaging with the helium-ion microscope: A review. *Beilstein Journal of Nanotechnology* **2021,** *12*, 1-23.

6. Lybrand, R. A.; Austin, J. C.; Fedenko, J.; Gallery, R. E.; Rooney, E.; Schroeder, P. A.; Zaharescu, D. G.; Qafoku, O., A coupled microscopy approach to assess the nano-landscape of weathering. *Sci. Rep.* **2019,** *9* (1), 5377.

7. Schlüter, S.; Eickhorst, T.; Mueller, C. W., Correlative Imaging Reveals Holistic View of Soil Microenvironments. *Environ. Sci. Technol.* **2019,** *53* (2), 829-837.

8. BRUAND, A.; COUSIN, I., Variation of textural porosity of a clay-loam soil during compaction. *European Journal of Soil Science* **1995,** *46* (3), 377-385.

9. Seah, M. P.; Green, F. M.; Gilmore, I. S., Cluster Primary Ion Sputtering: Secondary Ion Intensities in Static SIMS of Organic Materials. *The Journal of Physical Chemistry C* **2009,** *114* (12), 5351-5359.

10. Bich, C.; Havelund, R.; Moellers, R.; Touboul, D.; Kollmer, F.; Niehuis, E.; Gilmore, I. S.; Brunelle, A., Argon cluster ion source evaluation on lipid standards and rat brain tissue samples. *Anal. Chem.* **2013,** *85* (16), 7745-52.

11. Leefmann, T.; Heim, C.; Kryvenda, A.; Siljeström, S.; Sjövall, P.; Thiel, V., Biomarker imaging of single diatom cells in a microbial mat using time-of-flight secondary ion mass spectrometry (ToF-SIMS). *Org. Geochem.* **2013,** *57*, 23-33.

12. Heim, C.; Sjovall, P.; Lausmaa, J.; Leefmann, T.; Thiel, V., Spectral characterisation of eight glycerolipids and their detection in natural samples using time-of-flight secondary ion mass spectrometry. *Rapid Commun. Mass Spectrom.* **2009,** *23* (17), 2741-53.

13. Gilmore, I. S.; Aoyagi, S.; Fletcher, I. W.; Seah, M. P. G-SIMS-a powerful method for simplifying and interpretation of complex secondary mass spectrometry 2012.

14. Gardner, W.; Cutts, S. M.; Muir, B. W.; Jones, R. T.; Pigram, P. J., Visualizing ToF-SIMS Hyperspectral Imaging Data Using Color-Tagged Toroidal Self-Organizing Maps. *Anal. Chem.* **2019,** *91* (21), 13855-13865.

15. Madiona, R. M. T.; Welch, N. G.; Muir, B. W.; Winkler, D. A.; Pigram, P. J., Rapid evaluation of immobilized immunoglobulins using automated mass-segmented ToF-SIMS. *Biointerphases* **2019,** *14* (6), 061002.

16. Madiona, R. M. T.; Winkler, D. A.; Muir, B. W.; Pigram, P. J., Effect of mass segment size on polymer ToF-SIMS multivariate analysis using a universal data matrix. *applied surface science* **2019,** *478*, 465-477.

17. Lai, H.; Deng, J.; Liu, Q.; Wen, S.; Song, Q., Surface chemistry investigation of froth flotation products of lead-zinc sulfide ore using ToF-SIMS and multivariate analysis. *Sep. Purif. Technol.* **2021,** *254*, 117655.

18. Aoki, D.; Hanaya, Y.; Akita, T.; Matsushita, Y.; Yoshida, M.; Kuroda, K.; Yagami, S.; Takama, R.; Fukushima, K., Distribution of coniferin in freeze-fixed stem of Ginkgo biloba L. by cryo-TOF-SIMS/SEM. *Sci. Rep.* **2016,** *6*, 31525.

19. Fadel, A.; Lepot, K.; Nuns, N.; Regnier, S.; Riboulleau, A., New preparation techniques for molecular and in-situ analysis of ancient organic micro- and nanostructures. *geobiology* **2020,** *18* (4), 445-461.

20. Saatz, J.; Stryhanyuk, H.; Vetterlein, D.; Musat, N.; Otto, M.; Reemtsma, T.; Richnow, H. H.; Daus, B., Location and speciation of gadolinium and yttrium in roots of Zea mays by LA-ICP-MS and ToF-SIMS. *Environ. Pollut.* **2016,** *216*, 245-252.

21. Herrmann, A. M.; Ritz, K.; Nunan, N.; Clode, P. L.; Pett-Ridge, J.; Kilburn, M. R.; Murphy, D. V.; O’Donnell, A. G.; Stockdale, E. A., Nano-scale secondary ion mass spectrometry — A new analytical tool in biogeochemistry and soil ecology: A review article. *Soil Biol. Biochem.* **2007,** *39* (8), 1835-1850.

22. Musat, N.; Halm, H.; Winterholler, B.; Hoppe, P.; Peduzzi, S.; Hillion, F.; Horreard, F.; Amann, R.; Jorgensen, B. B.; Kuypers, M. M. M., A single-cell view on the ecophysiology of anaerobic phototrophic bacteria. *Proc. Natl. Acad. Sci. U. S. A.* **2008,** *105* (46), 17861-17866.

23. Stryhanyuk, H.; Calabrese, F.; Kummel, S.; Musat, F.; Richnow, H. H.; Musat, N., Calculation of Single Cell Assimilation Rates From SIP-NanoSIMS-Derived Isotope Ratios: A Comprehensive Approach. *Front Microbiol* **2018,** *9*, 2342.

24. Calabrese, F.; Voloshynoyska, I.; Musat, F.; Thullner, M.; Schlomann, M.; Richnow, H. H.; Lambrecht, J.; Muller, S.; Wick, L. Y.; Musat, N.; Stryhanyuk, H., Quantitation and Comparison of Phenotypic Heterogeneity Among Single Cells of Monoclonal Microbial Populations. *Frontiers in Microbiology* **2019,** *10*.

25. Malherbe, J.; Penen, F.; Isaure, M. P.; Frank, J.; Hause, G.; Dobritzsch, D.; Gontier, E.; Horreard, F.; Hillion, F.; Schaumloffel, D., A New Radio Frequency Plasma Oxygen Primary Ion Source on Nano Secondary Ion Mass Spectrometry for Improved Lateral Resolution and Detection of Electropositive Elements at Single Cell Level. *Anal. Chem.* **2016,** *88* (14), 7130-6.

26. Wagner, M., Single-cell ecophysiology of microbes as revealed by Raman microspectroscopy or secondary ion mass spectrometry imaging. *Annu. Rev. Microbiol.* **2009,** *63*, 411-29.

27. Xing, Z.; Du, C.; Zeng, Y.; Ma, F.; Zhou, J., Characterizing typical farmland soils in China using Raman spectroscopy. *Geoderma* **2016,** *268*, 147-155.

28. Luna, A. S.; Lima, I. C. A.; Rocha, W. F. C.; Araújo, J. R.; Kuznetsov, A.; Ferreira, E. H. M.; Boqué, R.; Ferré, J., Classification of soil samples based on Raman spectroscopy and X-ray fluorescence spectrometry combined with chemometric methods and variable selection. *Anal. Methods* **2014,** *6* (22), 8930-8939.

29. Stokes, D. L.; Wullschleger, S.; M., M.; Vo-Dinh, T. *Raman spectroscopy and instrumentation for monitoring soil carbon systems*; Department of Energy: 2003.

30. Cui, L.; Yang, K.; Zhu, Y.-G., Stable Isotope-Labeled Single-Cell Raman Spectroscopy Revealing Function and Activity of Environmental Microbes. Springer New York: 2019; pp 95-107.

31. Rebrošová, K.; Šiler, M.; Samek, O.; Růžička, F.; Bernatová, S.; Ježek, J.; Zemánek, P.; Holá, V., Identification of ability to form biofilm in Candida parapsilosis and Staphylococcus epidermidis by Raman spectroscopy. *Future Microbiol.* **2019,** *14*, 509-517.

32. Kumar, V.; Kampe, B.; Rösch, P.; Popp, J., Classification and identification of pigmented cocci bacteria relevant to the soil environment via Raman spectroscopy. *Environmental Science and Pollution Research* **2015,** *22* (24), 19317-19325.

33. Eichorst, S. A.; Strasser, F.; Woyke, T.; Schintlmeister, A.; Wagner, M.; Woebken, D., Advancements in the application of NanoSIMS and Raman microspectroscopy to investigate the activity of microbial cells in soils. *FEMS Microbiol. Ecol.* **2015,** *91* (10), fiv106.

34. Cui, L.; Yang, K.; Li, H. Z.; Zhang, H.; Su, J. Q.; Paraskevaidi, M.; Martin, F. L.; Ren, B.; Zhu, Y. G., Functional Single-Cell Approach to Probing Nitrogen-Fixing Bacteria in Soil Communities by Resonance Raman Spectroscopy with (15)N(2) Labeling. *Anal. Chem.* **2018,** *90* (8), 5082-5089.

35. Zhang, M.; Salje, E. K. H.; Farnan, I.; Graeme-Barber, A.; Daniel, P.; Ewing, R. C.; Clark, A. M.; Leroux, H., Metamictization of zircon: Raman spectroscopic study. *J. Phys.: Condens. Matter* **2000,** *12* (8), 1915-1925.

36. Sergeeva, A. V.; Zhitova, E. S.; Bocharov, V. N., Infrared and Raman spectroscopy of tschermigite, (NH4)Al(SO4)2·12H2O. *Vib. Spectrosc* **2019,** *105*, 102983.
